# A spatial regression analysis of Colombia’s narcodeforestation with factor decomposition of multiple predictors

**DOI:** 10.1038/s41598-023-40119-3

**Published:** 2023-08-18

**Authors:** Perla Rivadeneyra, Luisa Scaccia, Luca Salvati

**Affiliations:** 1 Dipartimento di Economia e Diritto, Universitá di Macerata, Macerata, Italy; 2 Dipartimento di Metodi e Modelli per l’Economia, il Territorio e la Finanza, Universitá di Roma Sapienza, Rome, Italy

**Keywords:** Environmental economics, Forestry, Environmental economics

## Abstract

In the current accelerated process of global warming, forest conservation is becoming more difficult to address in developing countries, where woodlands are often fueling the illegal economy. In Colombia, the issue of narcodeforestation is of great concern, because of the ramification of narcoactivities that are affecting forests, such as agribusinesses and cattle ranching for money laundering. In this study, we use spatially explicit regressions incorporating a factor decomposition of predictors through principal component analysis to understand the impact of coca plantations on global and local-scale deforestation in Colombia. At national level we find a positive and statistically significant relationship between coca crops and deforestation. At the regional level, in two out of four regions, it appears that coca is causing deforestation, especially in the Department of Northern Santander and on the Pacific coast. The spatial models used reveal not only a direct effect but also positive and significant spillover effects, in line with the conjecture that narcodeforestation is not only due to the quest for new areas to expand coca-cultivation, which would determine a loss of forest only in the municipality where coca cultivation increases, but also to the need to launder illegal profits, or create clandestine routes and airplane strips, which can affect forests also in nearby municipalities.

## Introduction

Containing deforestation is a critical environmental issue to be tackled in the short term, especially in the tropics. Forest loss is a complex phenomenon with multiple sources, which range from social, economic, and ecological nature. A detailed analysis of the spatial and temporal behavior of deforestation drivers is required to grasp the true causes and potential consequences of forest loss in a given region. This is notably important in Colombia, a country with a huge geographical, ecological, and cultural diversity, that preserves a considerable forest stock in Latin America. More than 52% of Colombia’s land is covered by forests, and 14% is classified as primary forest, the most biodiverse and carbon-dense form of forest. Colombia is also classified as the second most biodiverse country in the world holding 51,330 registered species until today^1^. However, deforestation is one of the main challenges to overcome in the short term if the country wishes to fulfill the requirements of the Paris Agreement. Under this legally binding international treaty, the 196 adopting countries are asked to put forward their best efforts to fight climate change through “nationally determined contributions” (NDCs). In particular, Colombia is called upon reducing greenhouse gases by 51% and black carbon emissions by 40% in 2030 compared to their 2014 levels^2^. Today, deforestation accounts for around a quarter of Colombia’s total emissions^3^. Thus, the country’s NDCs prescribe decreasing the deforestation rate to 50,000 ha/year and that of natural forest to 0 hectares/year by 2030. This objective is particularly ambitious, considering that Colombia is the 5th country in Latin America by deforestation rate, well ahead of Brazil or Mexico^4^. In Colombia half of the ecosystems are in a critical state of deterioration or a state of danger, moreover, in these endangered ecosystems more than a third of Colombia’s plants and 50% of its animals are at risk of extinction^5^. Similarly, indigenous and Afro-Colombian descendants, whose cultures and lifestyles are closely tied to these forested ecosystems, are also at risk of disappearing. Furthermore, according to Masiokas et al.^6^, the cryosphere of the Andes in Colombia has narrowed down by 62% in the last 50 years.

In addition to signing the Paris Agreement, Colombia has also adopted the United Nations’ Sustainable Development Goals (SDGs) as a framework for its development agenda. The country has committed to achieving all 17 goals and has aligned its National Development Plan with the SDGs. The goals are interconnected and integrated, and their achievement requires a comprehensive approach that addresses social, economic, and environmental issues. Despite the significant progress made toward achieving several of the SDGs, as said, the country continues to struggle with high levels of environmental degradation and deforestation, which pose a threat to sustainable development and the well-being of its citizens^7^.

In this framework, it is crucial to identify major causes of forest loss. Earlier studies identify and discuss the main drivers of tropical deforestation in the world. Among these factors, there are demographic drivers like population growth that push the colonization fronts, and economic elements like production boosts or extraction of raw material, infrastructure, and road development^8^. Moreover, environmental factors, including geology, topography, and soil type, as well as climatic factors, like drought and rainfall, are important natural determinants of deforestation^9^. In this sense, the relationship between the dry season and wildfires is especially conspicuous in the Amazon forest where scientists have observed that with the current climate change the length of the dry season has increased^8,10,11^, which has, in turn, led to an increase in wildfires. In the specific case of Colombia, the main drivers of deforestation are related to agriculture, fires, mining, cattle-grazing and population growth linked to clearance for government-planned settlement schemes^12,13^. Agribusinesses, particularly those involved in commercial crop production, have led to the conversion of forests into agricultural fields. Large-scale monoculture crops, such as palm oil, soybeans, and sugarcane, require vast amounts of land and often result in the clearing of forests to make way for these crops. Similarly, cattle ranching and the increasing demand for beef have driven the expansion of pastureland. This expansion is a major driver of deforestation, especially in regions such as the Amazon basin and the Colombian Orinoquia. In addition, the establishment of agribusinesses and large-scale cattle ranching operations often requires infrastructure development, such as roads, logging tracks, and processing facilities. These infrastructure projects can facilitate access to previously remote forest areas, leading to increased deforestation as more land becomes accessible for agricultural activities. Illegal activities, including illegal logging and land grabbing, also play an important role, and contribute to deforestation by clearing land without appropriate authorization or environmental regulations, encroaching upon protected areas, indigenous territories, and public lands^4,8,12,14^.

Several authors also consider the impact of coca cultivation on deforestation. As preliminary context, it is worthwhile noting that Colombia is the top producer of coca leaves in the world, the main input in the production of cocaine hydrochloride, an addictive psychostimulant drug whose use is illegal in most countries^15,16^. Colombia produces around 65% of the coca in Latin America, and with Peru and Bolivia, the three countries produce 98% of the coca worldwide^15,17^. In 2020 the production of coca leaves reached 143,000 hectares only in Colombia^18^. Coca production and its commercialization are deeply rooted in a complex web of social and economic factors. Colombia has a long history of coca cultivation, dating back to pre-colonial times when indigenous communities used coca leaves for cultural, medicinal, and ritual purposes. Over the years, factors such as economic marginalization, lack of state presence, armed conflict, and an exponentially increasing international demand have contributed to the expansion of coca cultivation. Nowadays, coca production often occurs in regions characterized by high levels of poverty and limited economic opportunities. Small-scale farmers may turn to coca cultivation as a means of survival and to escape poverty, since coca cultivation grants a higher income compared to other agricultural activities. Illegal coca trade provides significant economic incentives for farmers, middlemen, and criminal organizations involved in the production and distribution of cocaine. The high profitability of the drug trade has created a powerful economic force that fuels coca cultivation. The presence of illegal armed groups, including guerrilla organizations, paramilitary groups, and drug trafficking cartels, has also played a significant role in the dynamics of coca production. These groups have exploited coca cultivation as a source of revenue to finance their activities. The armed conflict in Colombia has further complicated efforts to address coca cultivation, as it has created challenges for state institutions and hindered alternative development initiatives.

The impact of coca crops on deforestation is complex because of the different actors involved, but also because of the indirect effects of the cultivation of coca. To refer to this complex phenomenon, the term *narcodeforestation* has been coined^19^, to indicate the forest loss determined not only by the direct effects due to the expansion of coca cultivation, which make growers move into more and more remote regions of Colombian forests in search of new fields to work, but also by indirect effects due to various drug baron activities (narcoactivities). For example, the need to launder illegal profits is a strong incentive for drug organizations to convert forests to agriculture (usually pasture or oil-palm plantation). Buying remote land and clearing it for farming or ranching activities allows dollars to be untraceably converted into private assets^20^. Another example of narcoactivity is provided by forest cutting to carve out clandestine routes and airplane strips for increased access to and through the jungles, spelling increased destruction for the already fragile ecosystems. It has been observed in Central America, especially in Guatemala, Honduras, Nicaragua, and Costa Rica, that narcotrafficking itself is a cause of deforestation^19,21,22^. However, this relationship has not been studied in Colombia, because coca plantations make it difficult to single out the effect of trafficking from crops. Central America is a corridor to get to the consumers, while Colombia is the factory. Therefore, the amount of forest loss due to the trafficking of illegal drugs in Colombia is unknown. In addition to narcoactivities, also certain policies to contrast coca cultivation are suspected to have increased forest loss^23^, such as aerial spraying with glyphosate, a measure intended for eradication that was suspended in 2015 but might be reconsidered in the future^24,25^.

Additionally, the long-lasting insurgency of *las FARC* in Colombia has linked forest dynamics to the guerrilla history process. Peace and war between the State and the armed groups have determined the level of forest clearance in Colombia. Since the peace treaty between *las FARC* and the State was signed in 2016, after 52 years of armed conflict, a drastic increase in forest loss occurred due to legal and illegal land grabbing of former *las FARC* occupied land^20^, converted into large scale cattle-ranching, coca cultivation or other illegal land markets. After the peace treaty, new fragmented organizations from *las FARC* began to take shape, and the COVID pandemic struck the cocaine market^26^, due to radical restrictions in national and international transportation. The price of cocaine has dropped due to hard lockdowns that have paralyzed transport flows worldwide^26,27^.

The present work aims at contributing to the literature about the impact of coca cultivation on deforestation rates. In particular, most of the existing literature considers the impact at the national level and does not investigate differences among regions. National-level analyses might oversimplify reality, leading to political responses that could be applicable according to local conditions in some parts of the territory but not in others. For example, Mendoza^28^ examines whether the effect of coca cultivation on forest loss is conditional on the level of Colombia’s internal armed conflict, but without accounting for possible regional variation in this effect. Similarly, Fergusson et al.^29^ consider illicit coca crops as one of the possible channels through which paramilitary forces affect deforestation, at national level. On the contrary, other studies focus on very specific and restricted areas of Colombia. For example, Anaya et al.^30^ consider the drivers of deforestation in the Pacific ecoregion, while the analysis in Hoffmann et al.^31^ encompasses a small portion of the Amazon region, mainly situated in the department Putumayo, near Colombia’s south-western border with Ecuador. An important exception is represented by Armenteras et al.^8^, who provide a comprehensive analysis, while recognizing the importance of accounting for Colombia’s biophysical, socioeconomic, and demographic heterogeneity, and explore the sub-national variation in deforestation dynamics and its drivers, within the country’s four main natural lowland regions. Armenteras et al.^8^, however, do not focus on the particular role played by coca cultivation, but simply consider illicit crops as one of the many factors affecting deforestation. In addition, it is important to note that the spatial structure of the data is not considered. Another important contribution that provides a wide-ranging analysis, while trying to balance it with Colombian internal heterogeneity and peculiarities, is that of Dávalos et al.^32^. Using logistic regression and accounting for spatial autocorrelation of the errors, they model the probability of forest conversion as a function of coca crops as well as other predictors, separately for the north, center and south part of Colombia. This country partition of convenience, however, does not necessarily fit biophysical, socioeconomic or demographic characteristics.

In addition to lacking national coverage that accounts for sub-national heterogeneity, current literature on the effect of coca cultivation on deforestation also reaches contrasting results, leaving space for further investigation. In Ref.^29,32^, for example, coca cultivation is not found to be a factor strongly influencing deforestation rates when controlling for other covariates. A significant effect is instead found in Ref.^12^ for the Andean region, and in Ref.^8^ for the Carribean and Orinoco regions, but not for the Amazonian region. Results might, though, be affected by the unrealistic assumption of spatially uncorrelated errors. Also, some of the literature examining the effects of coca cultivation on deforestation does not control for other contributing factors^30^ or account for just a very limited number of them^32^. Finally, most papers dealing with deforestation in Colombia and using the Global Forest Change Dataset^33^ set arbitrary forest threshold values to obtain forest maps. Often, these values are much lower^28,34^ than those recommended for tropical forests^35,36^, casting doubts on the reliability of the maps obtained.

In this work, we thus investigate the role of coca cultivation as a possible determinant of deforestation in Colombia and try to contribute to the current literature in different ways. Firstly, we perform the analysis at both national and sub-national levels, using cross-sectional data at the municipal level. In this way, we hope to provide results at a disaggregation level that enables a meaningful discussion about policy diversification patterns. Secondly, we propose a sub-national partition that, while preserving the natural characteristics of the country, also guarantees a reasonable number of municipalities to be included in each macro-area, to allow consideration of the spatial structure inherent in the observations. To achieve this, a K-means cluster analysis is employed to preliminary identify four homogeneous regions with respect to deforestation and coca cultivation levels, which are then slightly readapted to obtain well-defined regions, encompassing a sufficiently large number of municipalities. Clustering on the basis of the two variables of interest allows us to define a partition of the Colombian territory that is potentially different from the natural one and that better respects the heterogeneity of the relationship between coca cultivation and deforestation. Thirdly, to control for factors affecting deforestation other than coca cultivation, we consider a set of 44 control variables, including biophysical, anthropogenic, and socioeconomic variables. Up to our knowledge, no other study on the link between coca cultivation and deforestation has ever considered such a comprehensive set of covariates. Their inclusion helps prevent a possible omitted variables bias in the coefficient expressing the impact of coca crops on deforestation, but at the same time may engender multicollinearity issues, resulting in scarcely accurate estimates of this coefficient. To avoid multi-collinearity, a spectral decomposition based on principal component extraction is implemented to reduce the dimensionality of the predictors’ matrix, while retaining as much information as possible from the data. Fourthly, we relax the unrealistic assumption of independent errors, found for example in Ref.^8,12^ and we consider the spatial structure in the data. In doing so, we try to overcome the inadequacy of traditional regression models when the assumptions necessary for their implementation are no longer valid. An inadequacy that manifests itself by either impairing the OLS method (the coefficients will be estimated without bias but with less precision, and the tests will no longer have the usual statistical properties), or producing biased and inconsistent estimates of the coefficients. However, we do not consider spatial modeling merely as a way of dealing with the nuisance of spatially correlated observations, but first and foremost as a tool to unravel fundamental aspects of the phenomenon under study. In this perspective, and also to corroborate the robustness of the results, we compare different possible specifications of spatial models, such as the Spatial Lag of *X*, the Simultaneous Autoregressive, the Spatial Error, the Spatial Autoregressive Combined, the Spatial Durbin, the Spatial Durbin Error Model, and the General Nested models, allowing for both direct and spillover effects of coca crops on deforestation. Fifthly, we base our analysis on deforestation data based on the Global Forest Change Dataset but, unlike previous literature, we make use of a reasoned choice of the forest threshold, which accounts for the specific forest characteristics of the country.

We believe that the topic in this paper is of particular relevance in light of the need for a better understanding of the link between coca cultivation and deforestation, which can help in designing ad hoc policies to fight forest loss and climate change. Addressing these issues requires a comprehensive approach and our work suggests that in some areas, namely the Pacific and the Andean region, tackling deforestation necessarily involves curbing coca cultivation, through interventions that include sustainable rural development, poverty alleviation, and security measures.

## Data and methods

### Data

We use cross-sectional data at municipal level. The municipalities are obtained from the GADM database (version 3.6). The original 1065 municipalities are then aggregated according to the supplementary material of Mendoza^28^, ultimately reducing the number of municipalities to 1060. This allows for simplifying the indexing and enhancing the process of data matching and data fusion^37^, as the 1060 municipalities sub-division is the most widely used by national institutions.

We consider a set of 48 different variables, including deforestation and coca crops, which are the pivotal variables for our purpose. We include biophysical attributes, like slope and elevation, anthropogenic variables such as population density, wildfires, and remoteness (mean municipal pixels distance to the nearest road), and socioeconomic variables such as wealth distribution, added value created at the municipal scale, or the ratio of value added created by primary and secondary economic activities. All the above-mentioned variables have been shown to be correlated with forest loss either in Colombia or elsewhere. The database contains cross-sectional information of each variable mean for the case of variables that change over time, like deforestation, coca crops or precipitation, but we also have static variables like elevation. Moreover, variables that do not vary greatly over time are also referred to a single year, an example is the age structure of the population. Most of the variables are obtained from government sources, NGOs, and agencies. In Table S1 in SI, we display the reference year and the source of data, for each of the variables included in the analysis.

#### Deforestation data

Deforestation and coca crops are the focus of our study, therefore, we characterize them in more detail in Table 1. The deforested area is calculated from the rasters of the University of Maryland, namely the Global Forest Change Dataset (GFCD). GFCD has commonly been criticized for over-quantifying forest cover in different parts of the world, but it has also been demonstrated that the calibration through the forest cover percentage depends on the ecological zone treated. In the calculation of the forest cover in the GFCD data set, the canopy cover percentage is manually assigned by the researcher. The canopy cover threshold of 30% is widely used, however, the optimum threshold for the GFCD data is higher for denser forest canopy regions^35,36,38,39^. Lwin et al.^35^ determined that the accuracy of the forest cover in GFCD was dependent on the selection of the appropriate forest cover threshold. The authors analyzed the case of Malaysia, where the main forest cover type is tropical rainforest, and concluded that the threshold should be set according to the ratio of tropical rainforest and total area. However, for different ecological zones, different thresholds are needed to reach higher accuracy. In summary, where tropical rainforest is dominant, selecting a higher threshold is advisable. The optimal threshold at a national level was around 40% for Malaysia^35^, 70% for Gabon^39^ and 95% for Santa Catalina in Brazil^38^. As a result of the previous analyses and in consideration of the abundance of tropical forest in Colombia, we set the threshold at 70% in the present study. The decision to use this threshold is also guided by the comparison with the data provided by different sources, namely the Instituto de Hidrología, Meteorología y Estudios Ambientales (IDEAM) and European Space Agency (ESA)^36^.

**Figure 1:**
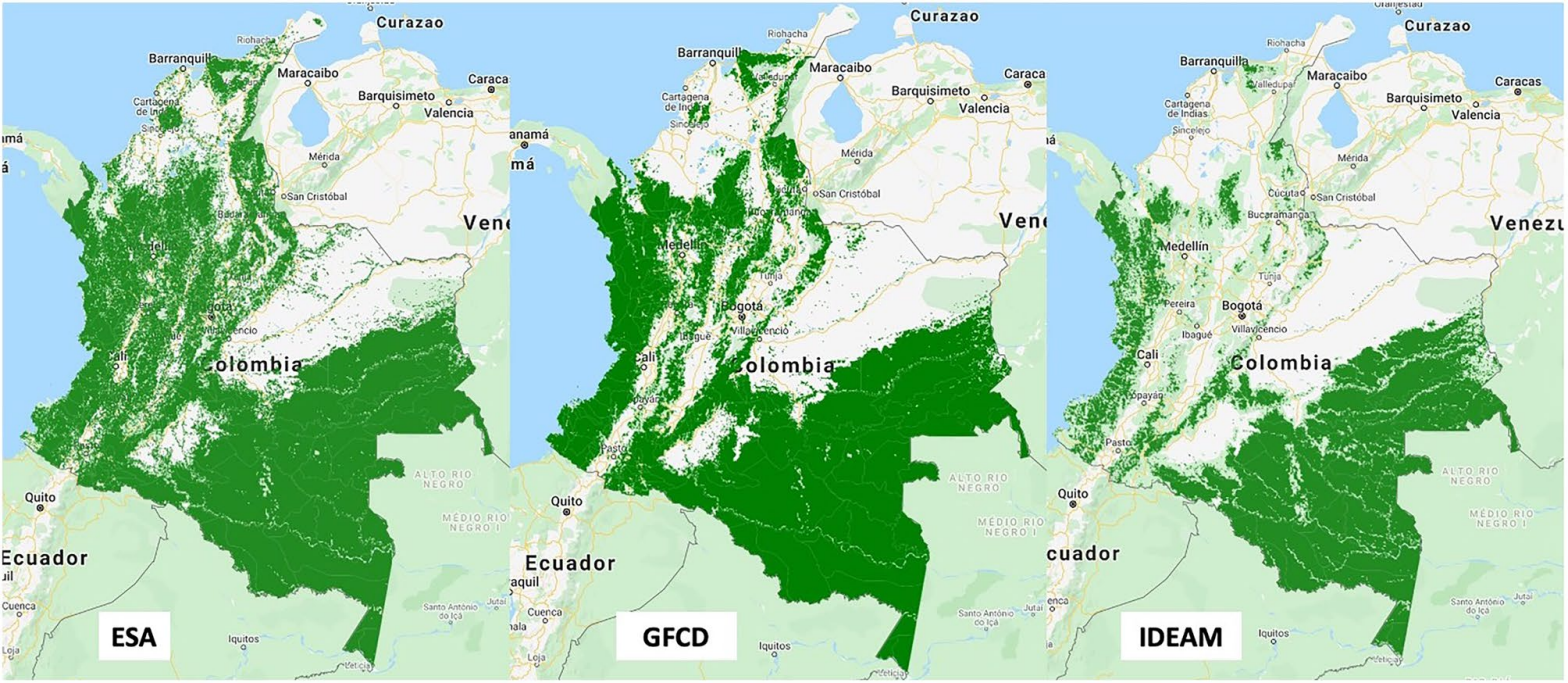
Comparison between ESA, GFCD and IDEAM forest cover (2017).

In the literature on Colombia’s narcodeforestation, many scholars use the IDEAM forest data set and a smaller portion uses the GFCD data set, whereas no one, up to our knowledge, has used ESA so far. GFCD’s data set goal is to quantify forest cover change and it takes the year 2000 as a baseline to confront the loss per year, while gain of forest cover was only monitored until 2012. GFCD inspects global Landsat data at a 30-meter spatial resolution to identify forest extent worldwide^33^. The IDEAM data set is collected and prepared by a Colombian institution that aims at quantifying forest cover, and has the advantage that the information obtained from the satellite and classification algorithms is debugged and analyzed by geographers who know the area. In this data set, the forest cover is pre-established, without the possibility to modify it, at 30%^40^. Also, the canopy height should be over 5 meters, which is a standard criterion compared to the other data sets. It is also a very easy-to-use file, it has only 3-pixel values, one for forest, another for non-forest, and one for no information, a relatively scarce value in this data set. The ESA data set is quite different from the previous two, since it does not allow to establish the forest cover like in GFCD, but has more options of canopy cover thresholds than IDEAM, it all depends on the type of land one wishes to include and the canopy associated to that land classification. In essence, the goal of the ESA data set is to classify the type of land into some 17 categories, which is quite different from IDEAM or GFCD, whose only goal is to identify forest and forest cover changes. These substantial differences in the goal and classification method between ESA and IDEAM/GFCD lead to differences in the results. Furthermore, GFCD uses high-resolution satellite imagery from Landsat, which also applies to IDEAM after 2010, while ESA uses Sentinel as its imagery source. According to Astola et al.^41^ Sentinel-2 is slightly better than Lansat 8 in forest variable prediction. Figure 1 provides a comparison between ESA, GFCD and IDEAM forest cover for the year 2017.

**Table 1:** Descriptive statistics on average annual deforestation area and coca crop extension, as percentage of municipality area, between 2000 and 2020.

Statistic	Deforestation	Coca crops
Null values	4	755
NA values	0	0
Min	0	0
Max	1.23	5.19
Median	0.06	0
Mean	0.12	0.06
Variance	0.03	0.07
Std. dev	0.16	0.27
Variation coef.	1.34	4.69
Skewness	2.89	10.03
Kurtosis	10.87	144.2

The main reason why we do not use IDEAM in our study is that it does not provide annual information within the time interval for which data on coca crops are available (from 2000 to 2020). IDEAM released intermittent rasters before 2012 and yearly information since 2013. Moreover, before 2007 the spatial resolution of the imagery obtained through MODIS was lower than that obtained from the Landsat satellite, which was used by IDEAM after 2010^40^.

**Figure 2:**
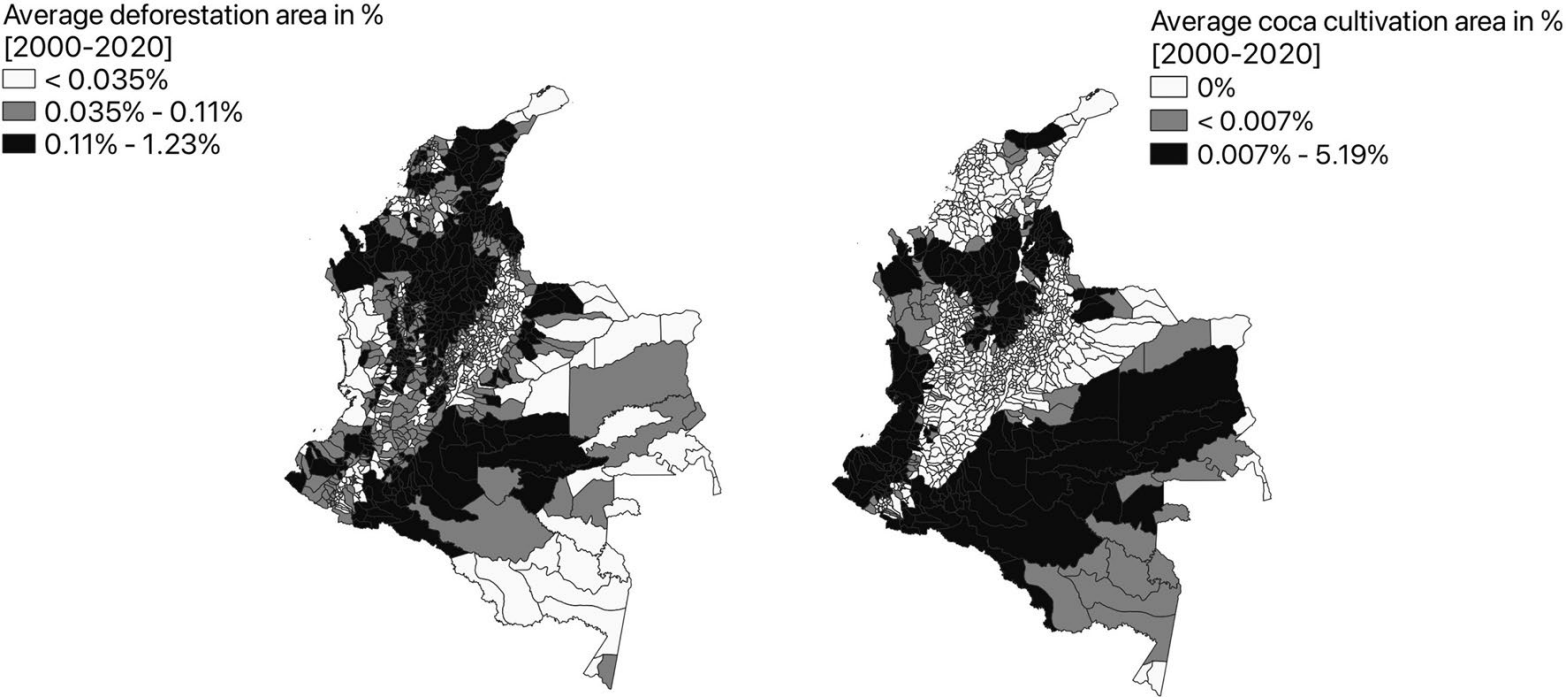
Average annual deforestation (left panel) and coca cultivation (right panel) area, as percentage of municipal area, between 2000 and 2020.

Table 1 provides some descriptive statistics on the average annual deforested area (as a percentage of the municipal area) in Colombia, during the period 2000–2020, on the basis of GFCD data. A graphical representation is, instead, given in the left panel of Fig. 2. The northwest of Colombia, which includes a considerable portion of the departments of Northern Santander, Santander, and Antioquia, shows a high mean deforestation area in the last 20 years. On the other hand, Caquetá, Guaviare, Meta, and Putumayo display large patch disturbances. These forested areas, highly valuable from a biodiversity perspective, are the main target of deforestation during the studied period.

#### Coca crops data

In this study, we use the data on coca crop extension published by the ODC (Observatorio de Drogas de Colombia). These data are produced through a multi-step process. Because of the illicit nature of the crops, various sources of information and validations are required. First, medium-resolution satellite imagery is obtained from three different satellites: Landsat, Sentinel, and Worldview II, depending on which satellite image encounters fewer clouds and offers, in general, a better view of the area. The images cover the whole extension of the Colombian mainland, except for the isles of San Andrés and Providencia (excluded from our analysis). Next, the images are pre-processed to enhance object detection. Later, the images are visually interpreted and, in this step, historical data and information from government sources that account for potential or verified coca crops are cross-checked with the images. Afterward, overflights on the coca crops detected through imagery take place for validation. Finally, the information is edited and published^42^.

**Figure 3:**
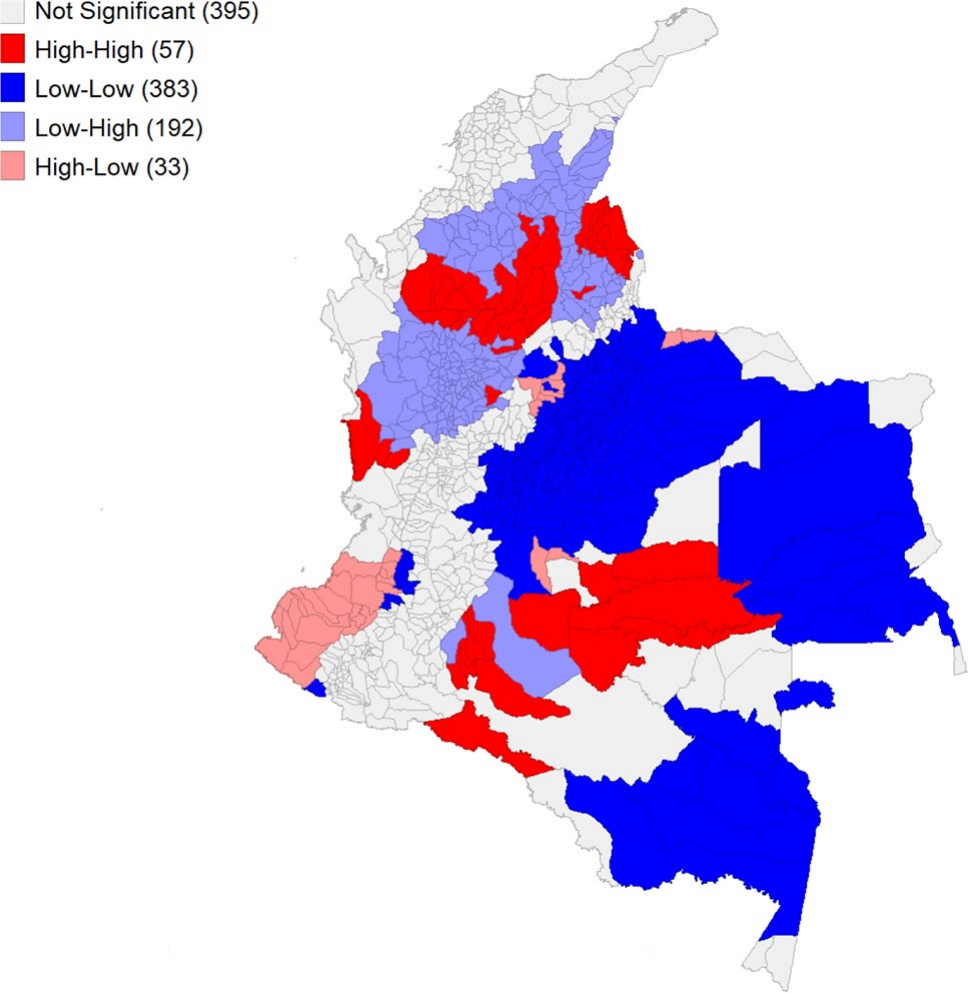
Bivariate local indicator of spatial association map.

We extract information on coca crops from 2000 until 2020, calculate the annual average, divide it by the area of the municipality and multiply it by 100. In this way, we obtain the annual average percentage of land covered by coca crops in each municipality. The result is shown in Fig. 2, right panel, while some descriptive statistics are provided in Table 1. From Fig. 2, we can identify 3 macro-regions of coca crops during the studied period. One is located in the north of the country, encompassing Chocó, Antioquia, Santander and Northern Santander. The second one is on the west coast, which comprises the unique biodiversity hotspot of Tumbes-Chocó-Magdalena, where concerns about the impact of deforestation are particularly relevant^43^. The third region, which extends over a very large area, wraps the strip that touches both the Andean foothills and the Amazon rainforest and includes the departments of Putumayo, Amazonia, Guaviare, Meta, and Vichada. To conclude the descriptive analysis of the main variables of interest, Fig. 3 shows a local indicator of spatial association (LISA) between deforestation and coca crops. In practice, it is a graphical representation of the local Moran I, with “High-High” (or “Low-Low”) values representing high (or low) deforested municipalities surrounded by high (or low) coca cultivated neighbors and “High-Low” (or “Low-High”) values representing high (or low) deforested municipalities surrounded by low (or high) coca cultivated neighbors. Out of the 1060 municipalities, 57 fall into the High-High group and 383 in the Low-Low group, which means that these data points have a positive spatial autocorrelation. A negative autocorrelation is instead found for 225 municipalities falling in the Low-High or High-Low groups. The LISA map suggests interesting locations of analysis and eventually clusters of both Low-Low and High-High alike values. A more wholesome analysis is offered in a multivariate context, and this is why spatial regression analysis is essential to gain more conclusive results on the studied relationship.

### Methodology

Spatial regression analysis is used to explore the relationship between coca cultivation and deforestation while allowing for the possible presence of spatial autocorrelation in the data. The fundamental interpretation of spatial autocorrelation is the existence of a spatial pattern. Positive spatial autocorrelation occurs when similar values are detected between the areal unit and its neighbors. Positive spatial autocorrelation does not mean that the observation and the neighborhood share positive values, instead, the observation and the neighbors show like-values, either positive or negative^44^. Conversely, negative spatial autocorrelation occurs when neighboring values are mutually dissimilar^45,46^. Spatial regression analysis is appropriate to investigate spatial patterns. Moreover, if spatial autocorrelation is present in the data and not specifically accounted for, the assumption of independent errors in OLS regression is violated, calling into question the validity of hypothesis testing procedures^47^.

**Figure 4:**
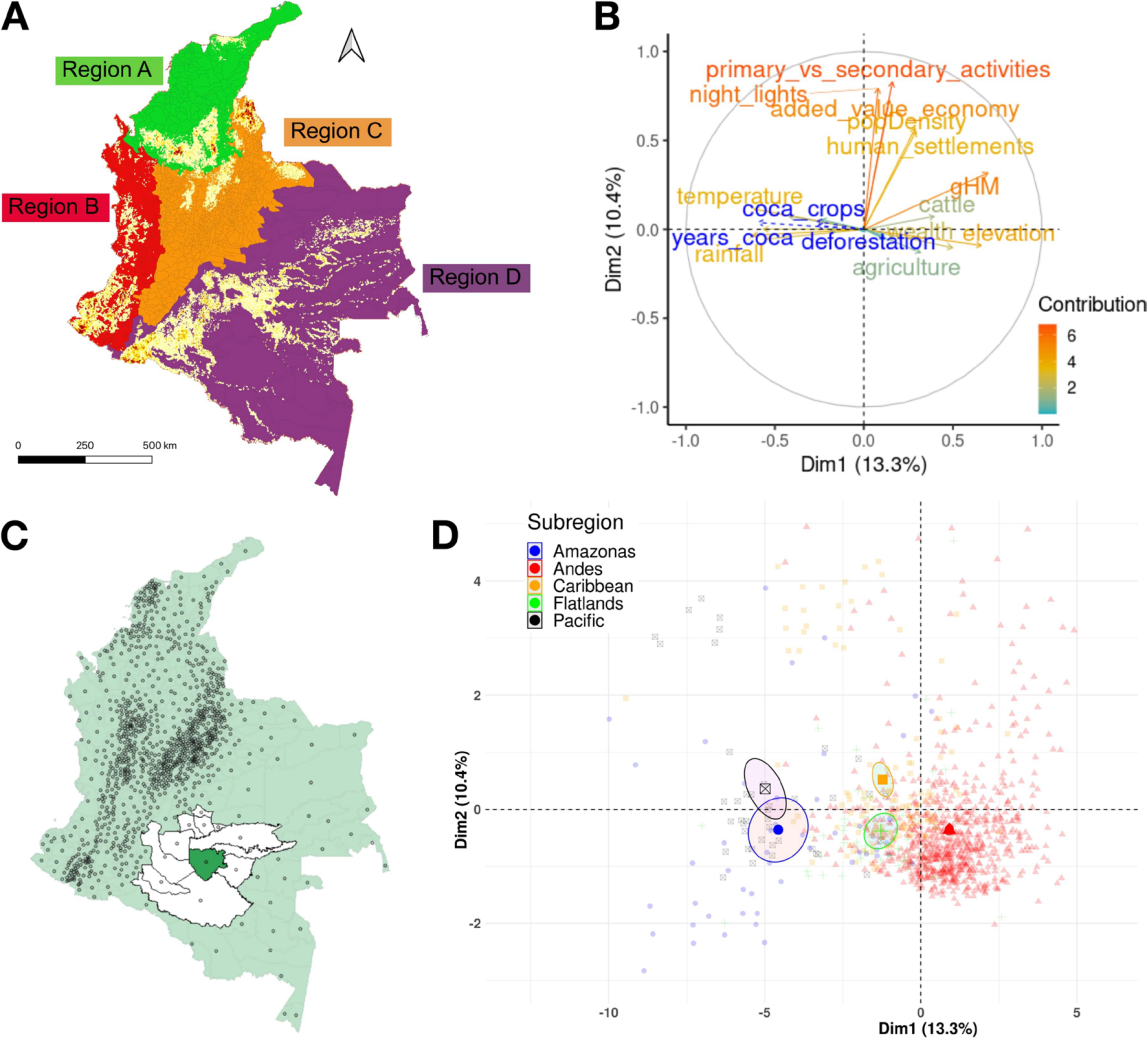
Preliminary results of the study: (**A**) K-means clustering of Colombian municipalities; (**B**) variable contribution to the first two principal components; (**C**) neighbors of the municipality of *Miraflores*, in the department of Guaviare, based on an inverse distance matrix with 200 km cut-off; (**D**) municipalities scores with respect to the first two principal components, colored according to the natural region to which they belong and with ellipses surrounding the 95% confidence interval for the centroid of the region.

Spatial weights matrices are the fundamental elements in regression models where representation of the spatial structure is needed. The spatial structure of the network contained in the weights matrix is determined and assumed *a priori*, which has often been criticized by economists, but was magnificently fenced by Corrado and Fingleton^48^, arguing, among other things, that weights matrices are “convenient, useful, and succinct representation[s] of spatial interaction[s], either in the form of endogenous or exogenous lagged variables, and/or as part of an explicit error process”. We use an inverse distance matrix, based on the Euclidean distance between centroids of the municipalities, with a cut-off of 200 kilometers. The coordinates of the centroids of the polygons in the system were originally in WGS84 (EPSG: 4326) coordinate system, later reprojected into WGS84/Pseudo-Mercator (EPSG 3857). This reprojection allowed conversion from degrees to meters of the coordinates. In the context of our study, the use of an inverse distance matrix with a cut-off is motivated by its ability to incorporate proximity-based interactions, capture localized spatial effects, improve computational efficiency, account for spatial autocorrelation, and provide flexibility in modeling spatial dependencies. In particular, by employing a cut-off distance, we focus the modeling efforts on capturing the most relevant local interactions while disregarding more distant and potentially less influential relationships. This helps to better represent the underlying spatial dynamics. In addition, the cut-off distance helps to balance between the very small polygons in the Andes and the very large ones in the Amazon. We can illustrate the neighborhood of the inverse distance criterion with a practical example. In Fig. 4C, *Miraflores* is a middle-size municipality in southern Colombia that has 11 municipalities considered as neighbors. Under this criterion, some municipalities in the Andean region have over 400 neighbors, because the municipalities are quite small, roads are more abundant and the population density is higher. Under this perspective, using different weights matrices, would likely produce awkward results. For example, a *K*-nearest neighbor matrix with *K* equal to a small value, would exclude relationships between very close municipalities of the Andean region, just because they are not neighbors of order *K*. On the other hand, choosing a larger value for *K* would determine very far municipalities in the Amazon to be related to each other. A similar reasoning would apply to queen contiguity-based matrices of order *K*. Despite the a priori validity of these arguments, also different weight matrices are considered and a sensitivity analysis is conducted.

The weights matrix accompanies either the dependent variable, the independent variable, the error term, or a combination of the previous terms, and this determines the differences between the various spatial models. A regression with spatial weights captures the amount to which the changes in an observational unit impact its neighbors, the so-called spillover effect^49^. Depending on the model, this effect can be measured at a global or local scale, as better explained later in this section. We consider seven spatial regression models, hence, the Spatial Lag of *X* (SLX) model, the Simultaneous Autoregressive (SAR) model, the Spatial Error Model (SEM), the Spatial Autoregressive Combined (SAC) model, the Spatial Durbin Model (SDM), the Spatial Durbin Error Model (SDEM), and the General Nested (GNS) model, which encompasses all previous models. The SLX model is estimated using Ordinary Least Squares, while the rest of the spatial models are estimated using Maximum Likelihood Estimation.

Using the notation proposed by Elhorst and Vega^50^, the GNS model has the following equation:

1 $$\begin{aligned} \begin{aligned} {\textbf{Y}} \,&=\, \rho {\textbf{W}} {\textbf{Y}} + \alpha \varvec{1}_N + {\textbf{X}} \varvec{\beta } + {\textbf{W}} {\textbf{X}} \varvec{\theta } + {\textbf{u}}, \\ {\textbf{u}} \,&=\, \lambda \textbf{W u} + \varvec{\varepsilon } \end{aligned} \end{aligned}$$

where ${\textbf{Y}}$ represents an $N\times 1$ vector consisting of one observation on the dependent variable for every unit in the sample, *N* being the number of these units, ${\textbf{X}}$ denotes an $N\times K$ matrix of explanatory variables, *K* being the number of these variables, $\varvec{1}_N$ is an $N\times 1$ vector of ones associated with the constant term parameter $\alpha$, ${\textbf{W}}$ is an $N \times N$ weights matrix that describes the spatial structure of the units in the sample^51^, and the $N\times 1$ vectors ${\textbf{u}}$ and $\varvec{\epsilon }$ denote the error terms of the model. It is assumed that ${\textbf{u}}$ follows a first-order spatial autoregressive process with spatial autocorrelation coefficient $\lambda$, and that $\varvec{\epsilon }$ has independent and identically distributed components, with zero mean and constant variance. Thus, ${{\textbf{W}}}{{\textbf{y}}}$ captures the endogenous interaction effects among the dependent variable, ${{\textbf{W}}}{{\textbf{X}}}$ the exogenous interaction effects between the independent variables, and ${{\textbf{W}}}{{\textbf{u}}}$ the interaction effects among the disturbance term of the different observational units. This model is the most general since it includes all types of interaction effects. The scalar coefficient $\rho$ is called the spatial autoregressive coefficient and reflects the spatial dependence inherent in our sample data, it measures the average influence on observations by their neighbors, while $\lambda$ is a scalar coefficient which captures the spatial correlation between errors, and $\theta$, just as $\beta$, represents a ${K} \times 1$ vector of response parameters. The OLS regression, which is obtained from the GNS model when all the spatial coefficients are set to zero, is used as a diagnostic tool for model specification and as a fundamental benchmark for spatial models. We calculate the Moran I test on OLS residuals to test the null hypothesis of no spatial autocorrelation, in which case the no rejection would imply the OLS model or non-spatial model to be adequate to explain the relationship between the variables^52^. On the other hand, upon rejection, we use Lagrange Multiplier (LM) tests for spatial dependence on OLS residuals to drive in the choice of an appropriate spatial model^45,53^. While the Moran test for spatial error autocorrelation is a general test, the LM tests are more specific. These statistics are meant to test for the presence of error dependence (LMerr, which in practice tests the null hypothesis that $\lambda =0$) and for the presence of a spatially lagged dependent variable (LMlag, which tests the null hypothesis that $\rho =0$). Significance of LMerr test points to a spatial error model, while the significance of LMlag test points to a spatial lag model. Robust variants also exist that test for the presence of one of the two forms of spatial dependence, in the presence of the other (RLMerr tests for error dependence in the possible presence of a missing lagged dependent variable, RLMlag the other way round). A portmanteau test, to test for both forms of spatial dependence, also exists (SARMA). While these statistics can be used on OLS residuals, with the specific aim of helping in the identification of the source of dependence, once a GNS model has been estimated, restrictions on its parameters can be tested using a robust t-test or F-test. These statistics can provide further evidence in favor of a specific spatial model. A graphical representation of the restrictions applied to the GNS model to obtain all the other spatial linear models is available in Fig. S12 of the SI. In Table 2, we instead provide the formula to compute direct and spillover effects, for all the models considered, as found in Ref.^50^, with $\mathbf {I_N}$ being an $N\times N$ diagonal matrix of ones, and we address the interested reader towards this reference for more details on the way these formulas are derived. In practice, a direct effect designates the effect of a change of a particular explanatory variable in a particular location *i* on the dependent variable of the same unit. A spillover effect, instead, designates the effect of a change of a particular explanatory variable in a particular location *i* on the dependent variable at a different spatial unit. Depending on the spatial model considered, the spillover effect can be local or global. The SLX and SDEM models determine local spillover, meaning that a change of a particular explanatory variable at a given location *i* will be transmitted to all those locations that, according to ${\textbf{W}}$, are connected to *i*. In contrast, the remaining models in Table 2 (except for OLS and SEM, which produce no spillover effects) determine global spillovers, so that a change of a particular explanatory variable at a given location *i* will be transmitted to all other locations even if unconnected with *i*.

**Table 2:** Direct and spillover effects corresponding to different model specifications.

	Direct effect	Spillover effect
OLS/SEM	$\beta _k$	0
SAR/SAC	Diagonal elements of $(\mathbf {I_N}-\rho {\textbf{W}})^{-1}\beta _k$	Off-diagonal elements of $(\mathbf {I_N}-\rho {\textbf{W}})^{-1}\beta _k$
SLX/SDEM	$\beta _k$	$\theta _k$
SDM/GNS	Diagonal elements of $(\mathbf {I_N}-\rho {\textbf{W}})^{-1}(\beta _k +{\textbf{W}}\theta _k )$	Off-diagonal elements of $(\mathbf {I_N}-\rho {\textbf{W}})^{-1}(\beta _k +{\textbf{W}}\theta _k)$

We use spatial regression both on a national and subnational scale. Colombia has, in fact, a rich diversity in biophysical, cultural, social, and economic terms, which could lead to significant heterogeneity in the impact of coca cultivation on deforestation. Therefore, dividing the country into macro-regions is essential to achieve a better understanding of the effect of coca cultivation on deforestation across the country. On the other hand, excessive fragmentation of the territory would reduce the number of municipalities in each region, undermining the possibility to use spatial models. On the basis of these considerations, we preferred not to adopt the partition into the five Colombian natural macro-regions, used for example in Ref.^8,12^. Firstly, this natural partition encompasses regions, such as the Pacific or the Amazonian, with such a small number of municipalities that do not allow for the use of spatial regressions. Secondly, it may not correspond to the partition defined by the heterogeneous geographical structure of the effect of coca cultivation on deforestation. Municipalities grouping based on geographical coordinates into north, center and south, as the one used in Ref.^32^, overcomes the first but not the second pitfall of the natural macro-regions.

For these reasons, we rely on cluster analysis to obtain macro-regions with sufficiently high internal size and homogeneity in terms of deforestation and coca cultivation. Their number should be adequately chosen to account for the spatially heterogeneous relationship between the variables of interest, while allowing for accurate estimation of this relationship. The aim is to provide insights to be used in planning a reasonable number of targeted policy actions. We believe that a partitioning of the national territory into four regions represents a good compromise in this respect, resulting, on average, in 265 municipalities per region. Then, to define appropriate macro-regions, we adopt a two-step process. The first step is a K-means clustering of the 1060 municipalities, using coca crops and deforestation as input variables. Using just these two variables to define similarity between municipalities is meant to create groups as internally homogeneous as possible in terms of the relevance of forest loss and coca cultivation, and, hopefully, in terms of the relationship between them. The choice of the clustering method is, instead, driven by the fact that the K-means algorithm is a popular and widely used technique that allows to specify the desired number of clusters in advance, thus providing flexibility in defining the desired level of granularity in the clustering results. In addition, it tends to produce equally-sized clusters, which meet our need to create sufficiently large groups. Then, in the second step, we slightly readapt the results to obtain visually well-defined regions. This is necessary in order to avoid lonely points, without neighbors, in the distance matrices of the macro-regions. Obviously, using K-means clustering is just one possibility of grouping municipalities and different techniques might be investigated. An interesting alternative consists of model-based clustering. In practice, instead of using cluster analysis prior to employing spatial models, one could consider using a spatial latent class model^54^, in which the variable of interest has cluster-specific spatial correlations and the discrimination rule for allocating the observations to the different classes is unobservable. In this setting, class-specific parameters and class memberships are estimated by means of EM algorithm. Such a modelization approach might though suffer from identification issues, meaning that multiple parameter values can produce similar model outcomes. This can result in non-unique solutions and make it difficult to estimate model parameters accurately. In addition, latent class models typically require larger sample sizes compared to simpler models due to the increased number of parameters. Insufficient sample size can result in unreliable parameter estimates and statistical tests with low power. In this regard, K-means clustering represents a viable and simple solution to cluster municipalities, as it does not require additional parameters to be estimated.

To control for factors affecting deforestation, other than coca cultivation, we consider many different variables of demographic, socioeconomic, anthropic, and biophysical nature, as described in Data section. We adopt Principal Component Analysis (PCA) to reduce the dimensionality of the predictors’ matrix and, thus, avoid multi-collinearity issues in the regression, while retaining as much information as possible from the data. In general terms, a PCA projects the original data into new variables, called Principal Components (PCs), that are orthogonal to each other and are ordered so that the first few retain most of the variation from the original variables. Thus, only the first few PCs are incorporated in the regression as control variables, avoiding multicollinearity issues. In this way, PCA helps delve into the data and to identify clusters and latent similarities between observations. We run one country-level PCA and use the results in the regional regressions because we privilege the space in the regression models to be explicit. The PCA gives us a comprehensive decomposition of the underlying factors that act at a national level, simultaneously reducing the clear redundancy of the data set as well as allowing us to aggregate the variables in fewer meaningful factors. In other words, PCA analysis represents the first filter at a national scale, while the regression represents the regional level, which becomes spatially explicit when working with spatial models at a municipality level (i.e. elementary analysis unit). All components from the PCA having an eigenvalue of 1 or greater are incorporated as control variables in the OLS and spatial regressions of deforestation on coca crops, at both national and sub-national levels. The only exception is region D (see Municipalitiy clustering section), where we retain a smaller number of components because of the reduced number of observations in this region. We allow 1 variable for each 10–15 observations^55^, though retaining only components with statistically significant coefficients. We underline that using PCA is just one possible way to handle multicollinearity, and alternative strategies might be considered. For example, regularization methods, such as ridge regression or lasso regression, which introduce penalties in the estimation process, can handle multicollinearity in an effective way, by shrinking the coefficients to provide more stable estimates. Partial least squares represent another possibility, somewhat related to PCA, as they also imply the construction of orthogonal components. However, unlike PCA, these components are obtained in such a way as to maximize the covariance between the predictors and the response variable. Both regularization techniques and partial least squares, though, do not easily allow us to distinguish between the predictor of interest (coca crops) and predictors that are simply included as control variables to account for potential confounding factors. In this regard, PCA provides a simpler solution in our context.

We use the **R** software for most of the analysis. In particular, to fit spatial models, we exploit the *spdep* package that contains all spatial linear model functions^56^. All the codes to replicate the results in this paper, as well as the municipal data set used, are provided in dedicated repositories. The interested reader can find the links to these repositories at the end of the SI.

## Results

### Municipalities clustering

Using the clustering strategy described in the "Methodology" section, we divide Colombia into four regions, as shown in Fig. 4A, that correspond broadly to the following geographical regions: (A) Caribbean/Sierra Nevada, (B) Pacific/Western Andean Foothills, (C) Andean Mountain Range, and (D) Amazonia/Eastern Andean Foothills/Orinoco. The four regions include, respectively, 196, 173, 613, and 78 municipalities. As explained in the Methodology section, this partition does not correspond to the one used in Ref.^8^, but in any case, it does not differ drastically from it. Our region A mostly matches the Caribbean natural region of Ref.^8^. It features diverse landscapes, including coastal plains, mangrove swamps, tropical forests, and mountainous areas, particularly in the Sierra Nevada de Santa Marta. It is home to a mix of ethnicities, with a significant Afro-Colombian population, but also indigenous people, and people of European and Middle Eastern descent. Agriculture plays a significant role in the region’s economy. The fertile coastal plains support the cultivation of crops such as bananas, plantains, rice, and tropical fruits. Fishing is also important, with communities relying on the sea for their livelihoods. The region’s coastal location facilitates trade and commerce, with ports and commercial centers supporting economic activities and international connections. Despite this, it also faces socio-economic challenges, including income inequality, poverty, and access to basic services and infrastructure in some areas. Our region A differs from the Caribbean region only for the inclusion of nearly the whole Bolivar department and the exclusion of the Santander department. These differences are motivated mainly by the coca cultivation patterns observed in these two departments. The Bolivar department has experienced some instances of coca cultivation in the past, however, this activity is not as prevalent or extensive as in other areas of Colombia, typically the Andean and Amazon areas. This substantial absence of coca crops is a common characteristic with the rest of Region A. On the other hand, mountainous terrain and favorable climate conditions in some parts of Santander make it suitable for coca cultivation. In this department, coca cultivation is also associated with illegal activities and the production of cocaine, posing challenges to local communities, the environment, and overall security in the area. In this respect, region A compared to the natural Caribbean region seems to better correspond to the coca cultivation patterns heterogeneity and appears to be a region where coca cultivation is very scarce.

Also Region B resembles closely the natural Pacific region in Ref.^8^ but includes in addition the western part of Andean Foothills. The region is renowned for its rich biodiversity and is considered one of the world’s most biologically diverse areas. It is home to lush tropical rainforests, mangrove swamps, and diverse marine ecosystems. The Chocó rainforest, which covers a significant part of the Pacific region, is one of the world’s wettest and most ecologically important rainforests. The region has a predominantly Afro-Colombian population, but several indigenous communities are also present. From a socio-economic point of view, it is characterized by high poverty rates, limited access to basic services, and economic disparities. Infrastructure development and job opportunities are relatively limited compared to other parts of the country. Agriculture and fishing are important economic activities in the region, with communities relying on subsistence farming, small-scale agriculture, and traditional fishing practices for their livelihoods. As for region A, coca cultivation is not a major concern in this region, except for the southwest part, namely the departments of Cauca and Nariño. Deforestation appears to be instead more important and particularly concentrated in the north and south part of the region. Similar levels of deforestation and coca cultivation are shared by the western Andean Foothills, which are, in fact, included in the region by the K-means algorithm, allowing to increase the number of municipalities, which is less than 30 in the natural Pacific region considered in Ref.^8^.

Compared to the natural Andean region in^8^, our region C is slightly smaller, excluding the western Andean Foothills (included in region B), part of the Bolivar department (included in region A) and the Putumayo department (included in region D), while including the whole Santander department. Despite these exclusions, region C remains the one with the largest number of municipalities. It is defined by its rugged and mountainous landscape, marked by high peaks, deep valleys, and fertile plateaus, encompassing a wide range of ecosystems and microclimates, including páramos (high-altitude grasslands), cloud forests, and fertile valleys. The region is known for its agricultural production and is considered the country’s “breadbasket”, with agricultural activities such as coffee, maize, potatoes, vegetables, and livestock farming. It also hosts major urban centers in Colombia, including the capital city, Bogotá, as well as Medellín, Cali, and others. These cities are hubs for commerce, industry, education, and cultural activities. Deforestation in region C is significant, but it is generally lower compared to other regions of the country, such as the Amazon and the Pacific regions. On the contrary, region C encompasses some of the areas where coca cultivation is more abundant, although the exact amount can vary over time and across different departments within the region. In particular, the department of Norte de Santander has historically been known as a significant area for coca cultivation. Its diverse topography, which includes mountainous areas and fertile valleys, provides suitable environmental conditions, while the proximity of this territory to the Venezuelan border makes it a strategic location for illicit activities, including drug trafficking.

Finally, region D includes both the Amazon and the Orinoco natural regions of Ref.^8^. Both of them are characterized by some large municipalities, which would prevent the use of spatial regression on the two regions taken individually. However, the two regions are not so different in terms of deforestation incidence and coca cultivation, as evidenced by the fact that the K-means procedure considers them within the same group. Both Amazonia and Orinoco are major contributors to the production of cocaine in Colombia. Amazonia’s vast rainforests, remote areas, and difficult terrain provide favorable conditions for coca production. Illegal armed groups and drug traffickers take advantage of the region’s isolated nature to establish coca plantations and illicit drug production facilities. Orinoco’s geographical features, including dense forests and river systems, also offer suitable environments for coca production. Similar to Amazonia, it is susceptible to drug-related activities due to its remote and hard-to-reach areas. In addition, both of them experience the highest deforestation rates in the country.

### Principal component analysis

We select 44 variables for the direct construction of the principal components and 3 quantitative supplementary variables, namely the average annual deforestation area, the average annual coca cultivation area and the number of years during which coca crops were observed in the municipalities. These supplementary variables are highlighted in blue in Fig. 4B. Also, we consider a qualitative supplementary variable, defining five natural regions. Both quantitative and qualitative supplementary variables are additional variables that are not used in the construction of the principal components but are projected onto the existing PCA model to provide additional information or context, and find patterns in the data through graphical visualization. All variables are standardized, which is a customary procedure before performing PCA, considering that this technique is sensitive to the variance of the original variables.

The first component extracts most of the variability in the data (13.3%). The first two components account jointly for 23.7% of the total variance. This relatively low percentage of explained variance is normal in a large data set with a high number of poorly correlated variables. We include 14 components in the regressions, which retain a cumulative variance of about 70%. We remark that, in this paper, PCA is implemented mainly as a variable reduction technique to obtain orthogonal factors to include as predictors in subsequent econometric modeling. The rationale is to avoid multicollinearity problems, while controlling for the effect of as many variables as possible and thus limiting the risk of an omitted variable bias in estimating the effect of coca crops on deforestation. In other words, principal components are merely used as control variables. For this reason, we limit ourselves to the interpretation of just the first two principal components, addressing the interested reader to Figs. S5 to S7 in the SI, where the original variables are plotted in the dimensional spaces defined by couples of principal components, for all the components included in the regressions.

The variables with a larger, positive loading with respect to the first component are the global human modification index (gHM), an aggregate measure of anthropogenic pressure on a landscape, elevation, wealth, and, to a minor extent, those accounting for the agricultural and cattle farming vocation of the municipality, while the variables with a large but negative loading are temperature and rainfall levels. Therefore, municipalities scoring high on this component are mountainous ones, with more temperate climates and lower rainfalls, and where the environment has been profoundly modified by people to produce wealth, mainly through primary activities. Examples of these municipalities are Bogotá, with a mean elevation of 2640 meters above sea level (m.a.s.l.). Medellín, with a mean altitude of 1495 m.a.s.l., and Cali, 1018 m.a.s.l. These are also the municipalities that yield the most to the national GDP, in the same order. On the other side, municipalities scoring low on the first principal components are, for example, Fuente de Oro in Meta, with an altitude of 359 m.a.s.l. and Fundación Magdalena (10 m.a.s.l.), the two municipalities that contribute the least to the national GDP^57^. In summary, the first principal component relates to morphological and climatic characteristics, compatible and favorable to (and thus correlated with) agriculture and economic growth. In this respect, the five Colombian natural regions, considered auxiliary variables, appear to be quite differentiated in terms of scores on this first component (Fig. 4D). At one extreme, we find the Andes region, marked by mountainous terrain and temperate climate conditions that make this area ideal for agriculture, particularly for crops like coffee. This advantage historically attracted settlers and contributed to the development of cities in this region and to their economic growth. At the opposite extreme, we find the Amazonas and Pacific regions, which share characteristics such as low elevation and economic challenges due to factors like inadequate infrastructure, limited access to resources, or conflict-related issues. These regions also register a significant presence of indigenous communities, which often experience high levels of poverty and income inequality. The Caribbean and Flatlands regions fall somewhere in between the two extremes. Characterized by relatively flat and low-lying terrains, and a tropical climate with distinct wet and dry seasons, they have fertile soils, ample water resources, and favorable conditions that make them suitable for cultivating a variety of crops. It is also interesting to note how the first principal component is negatively correlated with the three quantitative auxiliary variables. Coca cultivated area and its temporal persistence appear to be more relevant in areas with high humidity levels, adequate and evenly distributed rainfalls, and with warmer temperatures that contribute to faster growth and higher alkaloid content in coca leaves. At the same time, vulnerable and marginalized communities facing socioeconomic challenges, including lack of basic services, limited educational opportunities, and weak institutional support are more susceptible to engaging in illicit activities. Forest loss is also taking place mostly in remote, less developed and marginalized areas. As both forest loss and coca crops negatively correlate with the first principal component, controlling for its effect in regression becomes vital in order to avoid an omitting variable bias in estimating the effect of coca cultivation on deforestation.

With regard to the second principal component, the variables showing a larger positive loading are the ratio between primary/secondary activities and tertiary activities, the Night Light Index (which quantifies the amount and intensity of artificial light visible at night as observed from satellite imagery), the added value of the municipality, its population density, and the percentage of its area occupied by human settlements. In practice, the second component provides a synthetic measure of human activities and infrastructure, particularly the presence of urban areas and economic development. Natural regions appear to be much less differentiated with respect to this second component, while showing a large internal variability between municipalities. Within each region we can find from sparsely populated rural areas with scattered settlements to more densely populated and economically developed urban centers. Also, coca cultivation and deforestation appear to be much less correlated with the second principal component than with the first.

### Spatial modeling

Once an appropriate weighting matrix is selected, spatial models are specified and estimated at both national and regional levels. We stress that national results are simply obtained by considering all Colombian municipalities, independently of their grouping region. Thus, the findings at the country level are not affected by the particular division considered and are comparable with national results from existing literature about the effect of coca cultivation on deforestation. As mentioned before, we use the Moran I test as a diagnostic tool to determine whether a spatial structure is present in the data. For this purpose, the null hypothesis of randomness in the OLS residuals can be tested against the alternative hypothesis of spatial autocorrelation. Table 3 shows the results of this test at both the national and regional levels. The results shown make use of an inverse distance weights matrix with a cut-off of 200 km. While the choice of the type of weights matrix is motivated in the Methodology section, the selection of the cut-off value is based on both informed knowledge of the study area and literature scrutiny. In this regard, Ref.^58^ provides some rules of thumb to aid in the specification of weights matrices. In particular, he suggests applying a somewhat under-specified (fewer neighbors) rather than an over-specified (extra neighbors) weights matrix, as over-specification reduces the power of tests. The author provides both empirical and simulation proofs that are also well adapted to our experimental conditions, so we considered this rationale as particularly appropriate for our case. In accordance with this line of thinking we oriented our choice of the cut-off towards a reasonably small distance. In addition, we also followed Ref.^59^, who proposed to choose the weights matrix in order to maximize Moran’s coefficient. So, the final value of 200 km for the cut-off of the inverse distance matrix is selected as the one maximizing the national level Moran’s coefficient, out of a grid of proposed values.

From Table 3, all models exhibit spatial autocorrelation. The LM test can then be used to assist in selecting the appropriate specification of the spatial structure. However, using the LM test exclusively is not enough, and it should be considered jointly with the inferential results on the spatial regression coefficients^60,61^.

**Table 3:** Specification tests: two sided Moran I (*z*-scores are given in brackets) and Lagrange Multiplier test.

Statistic	National	A	B	C	D
Observed Moran I	$$8.11e-02{^{***}}$$ (17.64)	$$6.62e-02{^{***}}$$ (9.24)	0.076$${^{***}}$$ (8.25)	$$5.92e-02{^{***}}$$ (15.42)	0.25$${^{***}}$$ (5.18)
LMerr$$^1$$	$$215.26^{***}$$	$$21.50^{***}$$	$$19.87^{***}$$	$$121.58^{***}$$	$$17.20^{***}$$
LMlag$$^2$$	$$128.12^{***}$$	$$13.63^{***}$$	$$20.95^{***}$$	$$47.60^{***}$$	$$22.80^{***}$$
RLMerr$$^3$$	$$93.55^{***}$$	$$8.51^{**}$$	1.04	$$76.73^{***}$$	0.02
RLMlag$$^4$$	$$6.40^{**}$$	0.63	2.13	2.75	$$5.62^{**}$$
SARMA$$^5$$	$$221.66^{***}$$	$$22.13^{***}$$	21.99	$$124.33^{***}$$	$$22.83^{***}$$

Significance $$^{***}p$$-value$$\le 0.001$$, $$^{**}p$$-value$$\le 0.01$$, $$^{*}p$$-value$$\le 0.05$$.

$^1$ Simple LM test for error dependence.

$^2$ Simple LM test for a missing spatially lagged dependent variable.

$^3$ Robust LM test for error dependence.

$^4$ Robust LM test for a missing spatially lagged dependent variable.

$$^5$$ LMerr + RLMlag.

Considering the results of LM test in Table 3, the inference on the coefficients and looking for the most parsimonious model, we select a final model for each region. The main results for the final models are shown in Table 4 at the national and regional levels, respectively. Complete results on the eight models estimated for each region are provided in the SI (see Tables S2–S7). As already said, all the results presented are based on the use of an inverse distance weights matrix, with a 200 km cut-off. However, a sensitivity analysis has also been performed, using moderately different cut-off values, as well as different types of matrices, such as the queen contiguity-based spatial weighting matrix of order 1 and the *K*-nearest neighbor spatial weighting matrix, with various, small values of *K*. Regressions’ results (signs and significance of predictor coefficients) are reasonably comparable among the different weights matrix specifications.

**Table 4:** Final spatial regression results.

	National	Regions			
		A	B	C	D
	SDEM	SLX	SLX	SDEM	SLX
(Intercept)	0.116 (0.334)	0.016 (0.048)	0.122 (0.126)	0.220 (0.681)	$-0.063$ (0.079)
Coca crops	$$0.164^{***}$$ (0.028)	$$-0.049$$ (0.063)	$$0.249^{**}$$ (0.081)	$$0.303^{***}$$ (0.039)	$$-0.063$$ (0.11)
PC1	$$-0.317^{***}$$ (0.052)	0.252 (0.139)	$$-0.3$$ (0.215)	$$0.365^{***}$$ (0.074)	$$1.169^{***}$$ (0.226)
PC2	$$0.050^{*}$$ (0.026)	$$-0.060$$ (0.074)	$$0.203^{*}$$ (0.09)	$$-0.180^{***}$$ (0.045)	—
$$\mathbf {W\cdot u}$$	$$0.932^{***}$$ (0.028)	—	—	$$0.959^{***}$$ (0.027)	—
$${\textbf{W}}\cdot$$coca crops	$$0.784^{**}$$ (0.253)	$$0.466^{**}$$ (0.146)	$$2.372^{*}$$ (0.931)	$$-0.158$$ (0.368)	$$-0.358$$ (0.376)
$${\textbf{W}}\cdot$$PC1	$$0.583^{**}$$ (0.198)	0.171 (0.212)	$$-2.266^{*}$$ (0.076)	1.063 (0.909)	$$-1.872^{**}$$ (0.676)
$${\textbf{W}}\cdot$$PC2	$$-0.070$$ (0.337)	$$-0.173$$ (0.155)	$$2.543^{*}$$ (1.077)	$$-3.780^{***}$$ (0.909)	—
Adj. R$^2$	—	0.642	0.477	—	0.551
Num. obs.	1060	196	173	613	78
Parameters	33	—	—	33	—
$\sigma ^2$	0.517	—	—	0.454	—
Pseudo $R^2$	0.468	—	—	0.537	—
Wald test	$$1077.845^{***}$$	—	—	$$1247.482^{***}$$	—
Log.Lik(linear)	$-1207.901$	—	—	$-651.028$	—
Log.Lik(spatial)	$-1168.683$	—	—	$-633.612$	—
AIC (linear)	2479.803	416.393	464.376	1366.056	187.16
AIC (spatial)	2403.366	355.992	408.687	1333.223	174.29
LR test	$$78.437^{***}$$	—	—	$$34.833^{***}$$	—

Significance: $$^{***}p$$-value$$\le 0.001$$, $$^{**}p$$-value$$\le 0.01$$, $$^{*}p$$-value$$\le 0.05.$$

**Table 5:** Model comparison of the estimated direct and spillover effects of coca crops.

Model	OLS	SLX	SAR	SEM	SAC	SDM	SDEM	GNS	Mean	Std. dev
Direct effects										
National	$$0.23^{***}$$ (0.03)	$$0.16^{***}$$ (0.03)	$$0.23^{***}$$ (0.03)	$$0.19^{***}$$ (0.028)	$$0.19^{***}$$ (0.04)	$$0.19^{***}$$ (0.03)	$$0.16^{***}$$ (0.03)	0.19 (0.19)	0.19	0.03
Region A	$0.12^{*}$ (0.06)	0.11 (0.06)	0.11 (0.11)	0.06 (0.06)	0.07 (0.22)	0.01 (0.11)	0.01 (0.05)	0.01 (0.1)	0.06	0.05
Region B	$$0.24^{**}$$ (0.08)	$$0.25^{**}$$ (0.08)	$$0.28^{***}$$ (0.09)	0.24 (0.082)	$$0.25^{*}$$ (0.09)	$$0.3^{*}$$ (0.1)	$$0.25^{***}$$ (0.07)	0.3 (0.43)	0.26	0.03
Region C	$$0.32^{***}$$ (0.03)	$$0.32^{***}$$ (0.04)	$$0.27^{***}$$ (0.03)	$$0.32^{***}$$ (0.036)	$$0.3^{***}$$ (0.04)	$$0.31^{***}$$ (0.05)	$$0.3^{***}$$ (0.04)	$$0.3^{***}$$ (0.04)	0.31	0.02
Region D	0.1 (0.11)	-0.06 (0.11)	0.06 (0.1)	0.05 (0.1)	0.06 (0.1)	$-0.06$ (0.1)	$-0.1$ (0.1)	$-0.09$ (0.1)	0	0.07
Spillover effects										
National	—	$$1.06^{***}$$ (0.18)	$$1.07^{**}$$ (0.49)	—	0.73 (26.48)	5.88 (9.37)	$$0.78^{**}$$ (0.253)	2.71 (191.9)	2.01	2.05
Region A	—	$$0.47^{**}$$ (0.15)	0.14 (0.1)	—	0.09 (0.2)	2.46 (1.6)	$$1.88^{*}$$ (0.94)	2.33 (1.62)	1.2	1.1
Region B	—	$$2.37^{*}$$ (0.93)	1.14 (5.98)	—	0.45 (9.27)	5.11 (12.46)	$$2.35^{***}$$ (0.86)	4.95 (67.84)	2.72	1.93
Region C	—	$-0.084$ (0.29)	0.94 (1.83)	—	2.05 (11.43)	$-0.16$ (20.81)	$-0.16$ (0.37)	$-0.97$ (13.79)	0.21	1.1
Region D	—	$-0.36$ (0.38)	0.07 (0.18)	—	0.04 (1.38)	$-0.49$ (0.58)	$-0.38$ (0.31)	$-0.56$ (2.13)	$-0.27$	0.27

Significance: $$^{***}p$$-value$$\le 0.001$$; $$^{**}p$$-value$$\le 0.01$$; $$^{*}p$$-value$$\le 0.05$$.

Standard errors and p-values of the SAR, SAC, SDEM and GNS models are simulated values with an initial random value seed equal to 1.

We choose the SDEM for the National and Region C data. The SDEM, as well as the SLX and the SEM, is a local model, which is theoretically justified particularly in the case of the national-level data. The purpose of choosing a local model is to avoid looped effects captured by the ${{\textbf{W}}}{{\textbf{Y}}}$ vector, which could bias the spatial parameters and account for effects that are not in our defined neighborhood in ${\textbf{W}}$. In regions A, B, and D, we choose an SLX model, which is the simplest of all spatial models. For both the SLX and SDEM, the single municipality effects are captured by $\beta$ and the surrounding effects by $\theta$. In these models, $\beta _j$ is a non-spatial parameter that reflects the impact of the $j{th}$ variable at municipality *i* on the response at the same municipality *i* (direct effects), which is the usual interpretation in a linear non-spatial model, while the parameter $\theta _j$ reflects the surrounding effect on municipality *i* (indirect effect or spillover). Hence, a crucial element of SLX and SDEM is that there are no prior restrictions imposed on the ratio between the direct effects and spillover effects, which is a shortcoming of the SAR and SAC models, for which the ratio between the spillover and direct effects is the same for every explanatory variable, which is unlikely to be the case in many empirical studies. We estimate also the GNS model for completeness but do not consider it as a possible “best” model for any of the data sets, because it is often over parametrized and prone to overfitting. Similarly, the Durbin model has a global spillover specification which might be counter-intuitive in our study case. Besides, unless theoretically supported, global spillover specifications are difficult to justify and have been overused in applied studies^50^.

The national model has an estimated direct effect of coca crops on deforestation of 0.164, which is significant at the 1‰ level: an increment of one standard deviation in coca cultivated area in a given municipality is associated with an increase of 0.164 standard deviations of deforested area in that municipality. As shown in Table 5 this direct impact is quite stable among different model specifications, ranging from 0.16 in the SLX and SDEM models to 0.23 in the SAR and non-spatial model, with a mean of 0.19 and a between models standard deviation of 0.03. The coefficient of coca crops in our SAC specification (equal to 0.183, see Table S3 in SI) is also in line with those obtained by Mendoza^28^ under different spatial panel fixed effects SAC model specifications (values ranging from 0.106 to 0.271 in low conflict intensity municipalities and from 0.095 to 0.250 in high conflict intensity municipalities). Note that Mendoza uses a per-pixel forest cover threshold of 30% for the GFCD database, while we set it at 70%, and he does not provide direct effects but only coefficient values, so we can only compare these coefficients with those we obtain under the SAC specification^28^. Therefore, at least at the national level, results seem to be quite robust also to this threshold specification. Results are instead different from those by Armenteras et al.^8^, which use non-spatial generalized linear models to study the drivers of deforestation in Colombia. Armenteras et al. provide only the sign of the coefficients, not their magnitude, and in their national model, they find a positive coca crops coefficient, which is however not statistically significant. Coming to the spillover effect of coca-crops, this is positive and significantly different from zero in the chosen model SDEM, as well as in the SLX and SAR, with a slightly larger magnitude in these last two models. Under the SDM and GNS models, instead, the magnitude of the spillover effect is much larger but not significantly different from zero. These different results are, though, not surprising, as for the SDM and GNS models the spillovers come from both endogenous and exogenous interaction effects, whereas in the SLX and SDEM models they are only due to exogenous ones. In addition, as already said, SDM and GNS models imply global spillovers. So the fact that spillovers under these two specifications are not significantly different from zero, reinforces the belief that local spillovers are more appropriate in the present context. We underline that the presence of significant and positive local spillovers is in line with the assumption that narcodeforestation is not only due to the quest of new areas to expand coca-cultivation, which would determine a loss of forest only in the municipality where coca cultivation increases, but also to the need to launder illegal profits or create clandestine routes and airplane strips, which can affect forests also in nearby municipalities. Finally, the coefficient of the lagged error term is positive, significantly different from zero, and takes values close to one in all the specifications that include a lagged error term, testifying a positive correlation between the error terms of neighboring municipalities (see Table S3 in SI).

In Region A, the direct effect of coca crops is not statistically significant, in all the models considered. Therefore, for this region, the data do not seem to support the claim of an impact of coca crops on deforestation within municipalities. This result is in line with some previous findings. Dávalos et al.^32^ also conclude that coca cultivation is not associated with an increase in the probability of deforestation in the northern part of Colombia. On the contrary, Armenteras et al.^8^ find a significant and positive coefficient for illicit cultivation in their Caribbean region. Note, however, that their Caribbean region, with respect to our region A, includes a good portion of the Santander department, which is a department with a particularly strong association between coca cultivation and deforestation. See the discussion of the results for Region C for further details. It is interesting to note that region A also includes a part of the department of Antioquia, an area where the gHM index is high and the land used to grow coca is not always covered by forest, but already intended for other human uses, in most cases. For this reason, coca cultivation is not causing further deforestation in this area. The bivariate LISA map, being limited to the interaction of the two variables alone, provides results that can be somewhat in contrast with those obtained through multivariate regression. Nevertheless, there are positive and significant spillover effects under both the SDEM (at the 5% level) and the SLX (at the 1% level) model.

In Region B the estimate of the direct effect of coca crops on deforestation is quite stable among different models. For the SLX model chosen for this region, its value is 0.25, significantly different from zero at the 1% level. In addition, a positive and significant spatial local spillover can also be observed. Similarly, Dávalos et al.^32^ show that coca cultivation increases the probability of forest conversion in the Chocó department, included in our region B.

Also, region C has a positive, statistically significant and stable across models direct effect of coca crops. In particular, this is the region showing the highest values of this effect, immediately followed by region B. For the SDEM model the estimated direct effect is equal to 0.303 and highly significant. For this region, the bivariate LISA map pinpoints a large hotspot in Northern Santander. This is an area, as already said, where coca cultivation seems to strongly contribute to deforestation. For example, moving this hotspot to region A would make the direct effect of coca crops in this region positive and highly significant, providing results in accordance with those of Armenteras et al.^8^ for their Caribbean region. Dávalos et al.^32^ also conclude that coca cultivation increases the probability of forest conversion in the northern Andes (a zone roughly included in our region C), even if they do not find a significant association for the central region as a whole. In the same vein, Armenteras et al.^12^ found a positive and significant relationship between illicit crops and the probability of deforestation in the Andean region. Differently from previous regions, spillover effects seem to be not significantly different from zero in region C. Lastly, the spatial autocorrelation coefficient $\lambda$ has a value near 1 and is statistically significant in the spatial specification in which it is present (Table S6 in SI), implying that the error terms across neighboring spatial units are correlated.

In region D, neither the direct nor the indirect effects of coca crops appear to be significantly different from zero. Thus, data do not provide enough evidence to support the claim of an impact of coca crops on deforestation in Amazonia and Orinoco. This region has only 78 observations, for this reason, we removed non-significant principal components from the regression. In Amazonia and Orinoco, municipalities span over large territories, thus the lack of detail in the information may act as a source of errors, and the spatial models are probably capturing it. With respect to this area, Armenteras et al.^8^ found a non-significant relationship between illicit crops and deforestation in Amazonia, while they conclude in favor of a positive association between the two variables in Orinoco. In the Southern region, Dávalos et al.^32^ found that the probability of transition from forest to non-forest is found to increase significantly as coca cultivated area increases, and to decrease significantly as the distance from new coca crops increases. However, their Southern region encompasses only part of our Region D, while including portions of Region C and B, thus undermining results comparability.

## Discussion and conclusions

This study responds to the growing need to understand the main drivers of tropical deforestation, which are causing an alarming biodiversity loss worldwide, endangering half of Colombia’s ecosystems, impacting the monetary land resources, and therefore negatively impinging on the economy of the country^5,62^. In particular, the aim of this study is to assess if, and to what extent, coca crops contribute to deforestation. For this purpose, we used spatial models on cross-sectional municipality data, running regressions at both national and macro-regional levels, in order to account for possibly heterogeneous effects. Macro-regions were defined on the basis of coca crops and deforestation values, using the K-means method plus a slight readjustment to obtain four well-defined regions. A set of over 40 relevant control variables was considered and its dimensionality reduced by means of principal components analysis, while retaining more than 70% of the original variability in the data set.

The novelty of our approach, in comparison with previous studies, is manifold. Firstly, rather than relying on predefined and not necessarily suitable grouping of municipalities into a north-center-south classification or similar geographical classifications, we selected macro-regions through an unsupervised machine learning method of clusterization, thus defining more homogeneous groups of municipalities with respect to the two variables of interest. Secondly, to compute forest loss extension, we accurately chose the threshold for the GFCD data and set this value to 70%, basing our choice on the consideration of the predominant forest type in Colombia and following the guidelines in^35,36^. Thirdly, we gathered a relevant number of variables from very different sources, creating - up to our knowledge - the largest database of control variables ever used in studies on deforestation in Colombia. Finally, we compared seven different spatial models to assess the stability of direct and spillover effects of coca crops on deforestation.

Our results indicate that the presence of coca cultivation increases notably the extent of deforestation in Colombia. This is particularly true in two regions that include two pivotal biodiversity hotspots in the Andes and the Pacific Coast. The other two regions, Amazon/Orinoco and Caribbean/Sierra Nevada did not show a significant direct effect of coca crops on forest loss, although significant spillover effects could be present in the Caribbean/Sierra Nevada region.

In the current debate on the negative environmental outreach of coca cultivation, there is a general acceptance that an effect on deforestation does exist. In this study, we showed that this impact is not negligible, and is amplified by spillover effects. These turned out to be particularly relevant, an aspect that surely deserves further investigation. A possible cause can be ascribed to trafficking, which is the second step in the commercialization of this plant-based drug. Even if not yet proven, probably coca trafficking is driving deforestation within the producing countries. What is certain is that this is happening in transiting countries, like Central America, where the effect has been singled out^19,21,63^.

We believe that this work provides important findings for policymakers and civil institutions that are concerned with deforestation patterns and want to pinpoint the leading causes in Colombia. To mitigate deforestation it is crucial to address the underlying drivers and our work supports the claim that coca cultivation is one of them. Reducing coca crops can help in preserving forests and, as McSweeney et al.^19^ clearly state, “drug policy is conservation policy”. Obviously, this is not a simple task and there is not a unique and permanent solution. The forces funneling coca-related activities and deforestation are changing constantly: the peace treaty, the COVID pandemic, the formation of new paramilitary groups, or the 2021 protests act as enhancers or diminishers of deforestation and coca cultivation. This was manifest with the peace treaty of 2016 that represented a watershed in the trend of deforestation and coca cultivation in the country. During conflict, many remote areas were occupied by the guerrilla, and this had the side effect of stunningly protecting forests from private interests and containing the agricultural frontier. Therefore, when this force disappeared, land grabbing and private interests took over the liberated lands, and the peace treaty unintentionally determined an impressive acceleration in deforestation. This was also fueled by the rapid increase in coca cultivation, which followed the peace treaty, and which was partly due to the failures in the implementation of adequate substitution programs. So far, drug policies have failed to reduce coca cultivation and trafficking, sprawling on the contrary violence and corruption. This was also due to the fact that drug policies are wrongly focused only on the supply-side through interdiction and eradication, while a more unwavering solution would be to focus on the demand-side^19^.

In conclusion, the extremely varied and disparate sources of our seemingly large data set imposed a challenge in terms of data matching and data fusion, whereas our ultimate goal was to obtain a record with comparable, meaningful, clean and consistent information. However, the fact that our data set contains more variables than other comparable studies is an advantage in terms of robustness of the spatial regressions, but is also a disadvantage as it represents a source of multiple and varied types of intrinsic errors that were patent in some of the variable’s sources. Colombia is in the process of constructing reliable statistics and data, but this is just happening in these latest years, hence, historical data on economic and social aspects, in the best case scenario, date back to the early 2000s. Moreover, data are not collected regularly or with a consistent methodology through the years, and in many cases the opposite is the rule, meaning that data are found in intermittent years and changing methodologies are quite common^64^. Considering that the quality and span of historical data are likely to enlarge and improve in the upcoming years, monitoring of the forest’s state as well as future multivariate regressions to understand the etiology of forest loss should profit of this increased accuracy. In connection with these data availability and reliability worries, we posed in this work the first concerns regarding the most suitable data source, between GFCD, ESA or IDEAM, to estimate deforestation rates in Colombia, and we tried to provide a more reasoned choice of the threshold used for the GFCD^35,36^.

The improvement in data quality and availability also lays the foundation for future research. In particular, endogeneity and reverse causality are important issues to consider when studying the impact of coca cultivation, or other factors, on deforestation. We tried to deal with these issues controlling for as many confounding factors as possible and through robustness checks, by employing alternative model specifications to assess the potential impact of unobserved confounding on the estimated effects. Better data availability would pave the way to more sophisticated techniques. For example, the use of instrumental variables would help in isolating the exogenous variation in the endogenous variable and provide a way to estimate causal effects. Valid instruments are though required. Accurate temporal data with a sufficient spatial disaggregation would allow employing fixed effects models, which control for time-invariant unobserved factors at the individual spatial observation level, mitigating endogeneity and isolating within-observation variations. Also, space-time analysis would enable to examine the temporal order of events and incorporate lagged variables to account for the potential delay in the causal relationship, thus helping to address reverse causality by considering the precedence of cause and effect.

### Supplementary Information

Supplementary Information.

## Data Availability

The datasets used and/or analyzed during the current study are available at the following link: https://www.kaggle.com/datasets/perlarivadeneyra/dataset.
