# Supplementary material for: A spatial regression analysis of Colombia’s narcodeforestation with factor decomposition of multiple predictors
Source: Sci Rep. 2023 Aug 18;13:13485. doi: 10.1038/s41598-023-40119-3 (PMC10439211; doi:10.1038/s41598-023-40119-3)
Supplement: Supplementary file 1 — Supplementary Information. [file 41598_2023_40119_MOESM1_ESM.pdf]

Supplementary Information to: A Spatial Regression Analysis of Colombia's Narcodeforestation with Factor Decomposition of Multiple Predictors

Perla Rivadeneyra[1,*], **Luisa Scaccia**[1], **and Luca Salvati**[2]

[1]Dipartimento di Economia e Diritto, Università di Macerata
[2]Dipartimento di Metodi e Modelli per l'Economia, il Territorio e la Finanza, Università di Roma Sapienza
[*]Corresponding author: perlarivadeneyra@gmail.com

Table S1. Variables description, source of data and reference period

Identifier	Description	Source	Year(s)
PDET	Programa de Desarrollo con Enfoque Territorial	ArcGIS data base	2019
FARC07_16	FARC presence during 2007-2016	Misión de Observación Electoral	2007-2016
Wconflict	Victims of armed conflict	Centro Nacional de Memoria Histórica	2010-2016
mining	Mining area in percentage	Tierra Minada	2018
national_parks	National park area in percentage	Environmental Research Systems Institute (ESRI) Colombia	2020
other_parks	Regional and other typologies of parks	Environmental Research Systems Institute (ESRI) Colombia	2021
afrocolombian _protected_area	Percentage of afro-colombian territories on total area	Instituto Geográfico Agustín Codazzi	2018
elevation	Elevation mean	Zenodo	2017
elevstdev	Standard deviation of the elevation	Zenodo	2017
night_lights	Sum of night light events	NASA's Earth Observatory	2016
indigenous _protected_area	Percentage of indigenous reserves area on total area	Instituto Geográfico Agustín Codazzi	2018
wealth	Relative Wealth Index predicts the relative standard of living [**?**]	United Nations Office for the Coordination of Humanitarian Affairs (OCHA)	2021
added_ value_economy	Added value of the municipality	Departamento Administrativo Nacional de Estadística	2018
primary_vs_ secondary_activities	Primary and secondary activities as a ratio of tertiary activities	Departamento Administrativo Nacional de Estadística	2018
defense	Military and police stations per 1000 inhabitants	Datos Abiertos Gobierno de Colombia	2016
agriculture	Hectares for agricultural use as percentage of the total area	Environmental Research Systems Institute (ESRI) Colombia	2013- 2014
years_coca	Years of coca crops (permanence of coca crops)	Observatorio de Drogas de Colombia	2000-2020

Table ?? continued: Variables description, source of data and reference period

financial_ institutions	Financial institutions per 1000 inhabitants	United Nations Office for the Coordination of Humanitarian Affairs (OCHA)	2020
popDensity	Population density	NASA's Earthdata	2020
voting_turnout	Participation in the elections of 2018 as percentage of the total population	Departamento Administrativo Nacional de Estadística	2018
duque	Percentage of total voters of Iván Duque (right-wing candidate)	Departamento Administrativo Nacional de Estadística	2018
mean_youth _age_of_man	Man between the age of 15 to 24 years old	Departamento Administrativo Nacional de Estadística	2017
early_adults_age	Man between the age of 25 to 40 years old	Departamento Administrativo Nacional de Estadística	2017
women2017	Female population percentage	Departamento Administrativo Nacional de Estadística	2017
TFR	Total fertility rate	Departamento Administrativo Nacional de Estadística	2017
internal_ displacements	Internally displaced persons	United Nations Office for the Coordination of Humanitarian Affairs (OCHA)	2000-2014
private_property	Private properties for agricultural use	Environmental Research Systems Institute (ESRI) Colombia	2013- 2014
rented_property	Rented properties for agricultural use	Environmental Research Systems Institute (ESRI) Colombia	2013- 2014
usufruct_property	Usufructed properties for agricultural use	Environmental Research Systems Institute (ESRI) Colombia	2013- 2014
collective_property	Collective property for agricultural use	Environmental Research Systems Institute (ESRI) Colombia	2013- 2014
gHM	Global Human Modification Index (cumulative measure) [?]	Google Earth Engine Repository	2016
remoteness	Mean municipal pixels distance to nearest road	United Nations Office for the Coordination of Humanitarian Affairs (OCHA)	2020
water_capacity	Water holding capacity of soil, mean value	Zenodo	1990-2020

Table ?? continued: Variables description, source of data and reference period

WCstdev	Water holding capacity of soil, standard deviation	Zenodo	1990-2020
health_ infrastructure	Hospital infrastructures per 1000 inhabitants	Departamento Administrativo Nacional de Estadística	2021
poverty	Global Multidimensional Poverty Index (MPI)	United Nations Office for the Coordination of Humanitarian Affairs (OCHA)	2021
rainfall	30-year precipitation mean	Zenodo	1990-2020
cattle	Hectares intended for breeding of adult livestock	Departamento Administrativo Nacional de Estadística	2018
wildfires	30- year mean wildfires as percentage of total area	NASA	1990-2020
apersion_ glyphosate	Glyphosate aspersion as percentage of total area	Observatorio de Drogas de Colombia	2000-2015
temperature	30-year temperature mean	Zenodo	1990-2020
temperature_std	30-year temperature standard deviation	Zenodo	1990-2020
human_ settlements	Settlements as percentage of total area	European Space Agency (ESA)	2001-2020
palm_oil	Palm oil as percentage of total area	Google Earth Engine Repository	2019
cropland	Cropland as percentage of total area	European Space Agency (ESA)	2001-2020
other_ natural_land	Natural -non-forest- land percentage	European Space Agency (ESA)	2001-2020
coca_crops	20-year coca crop mean as percentage of total area	Observatorio de Drogas de Colombia	2000-2020
deforestation	GFCD’s 20-year mean deforestation as percentage of total area	University of Maryland	2000-2020

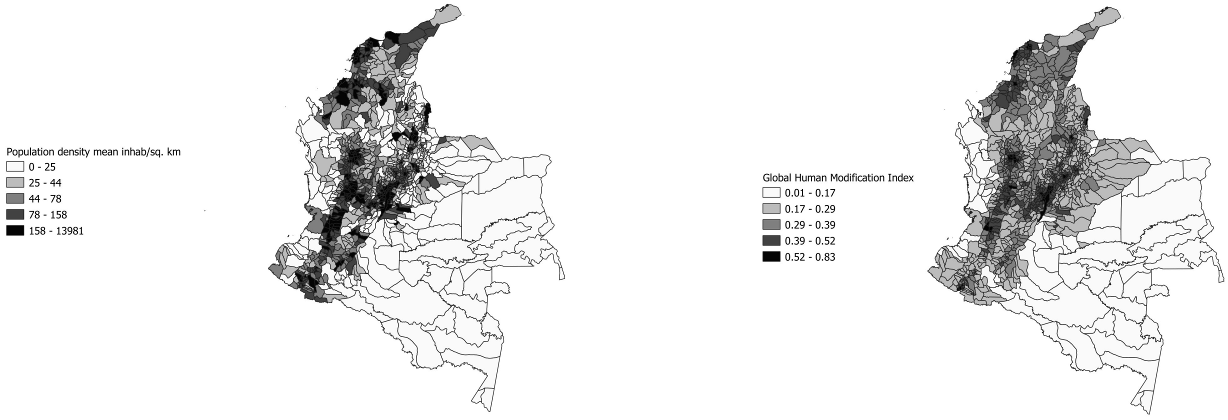

(a) **(b)**

Figure S1. (a) Population density map (b) Global Human Modification Index map.

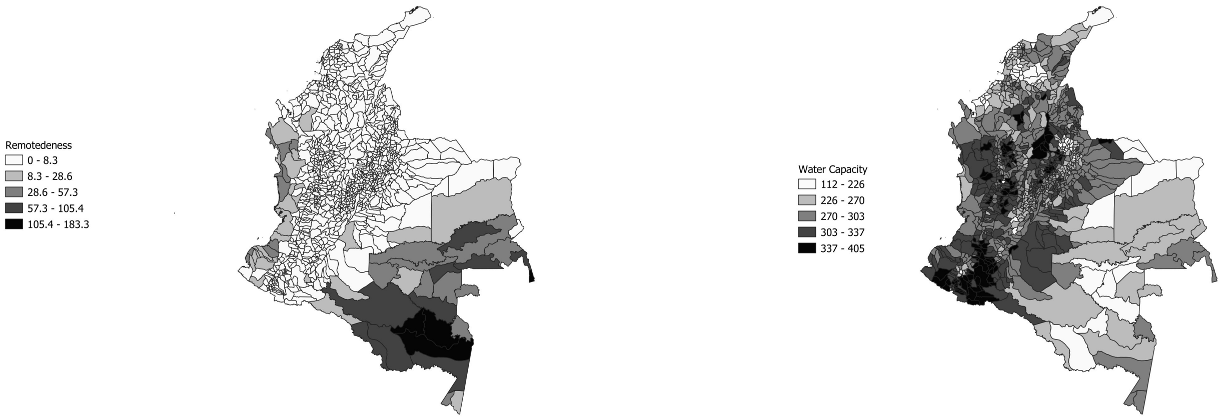

(a) **(b)**

Figure S2. (a) Remoteness map (b) Soil water capacity map.

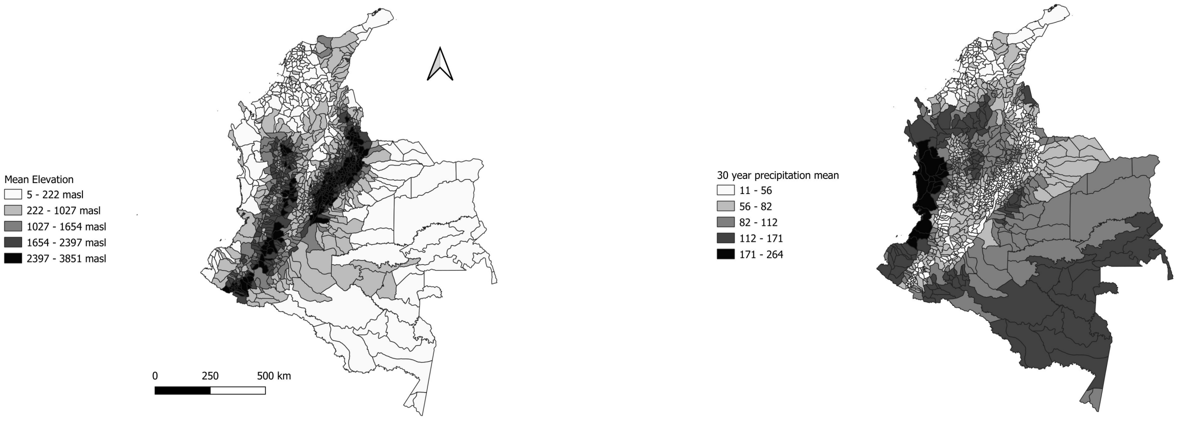

(a) **(b)**

Figure S3. (a) Elevation mean map. (b) 30 year precipitation mean map.

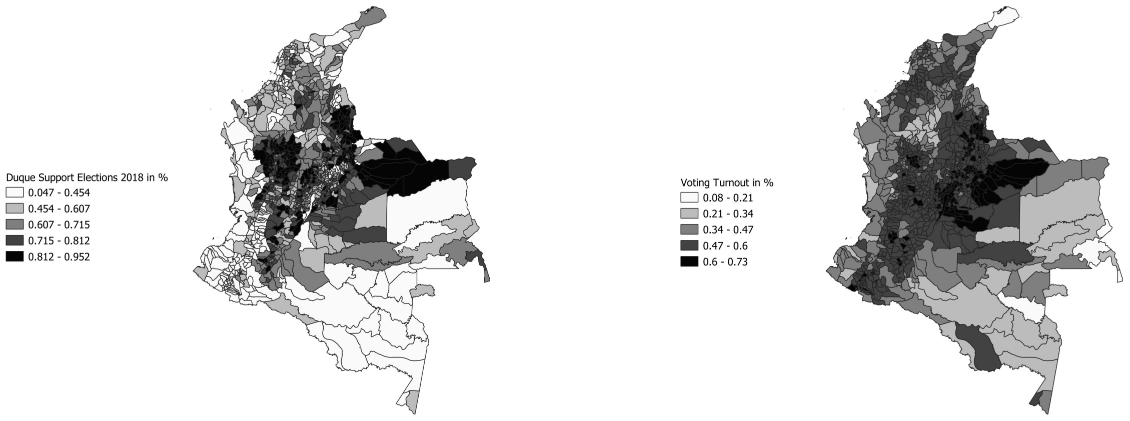

(a) **(b)**

Figure S4. (a) Map of duque supporters in % (b) Map of voting turnout in % (elections 2018).

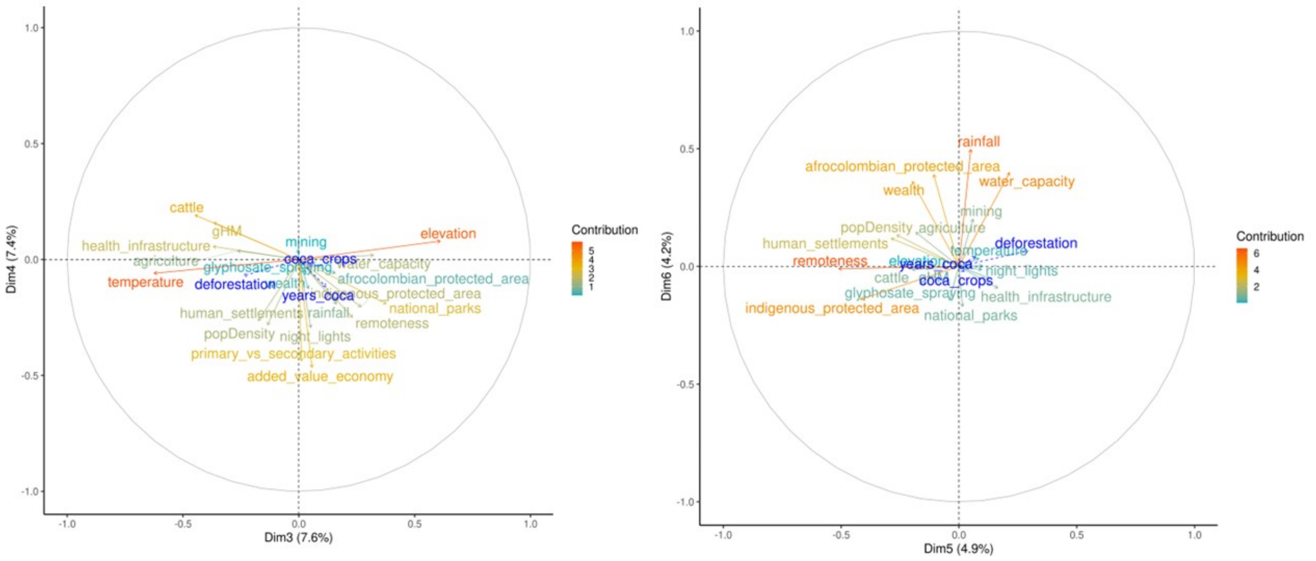

Figure S5. Variable contribution to dimension (left) 3 and 4 and (right) 5 and 6.

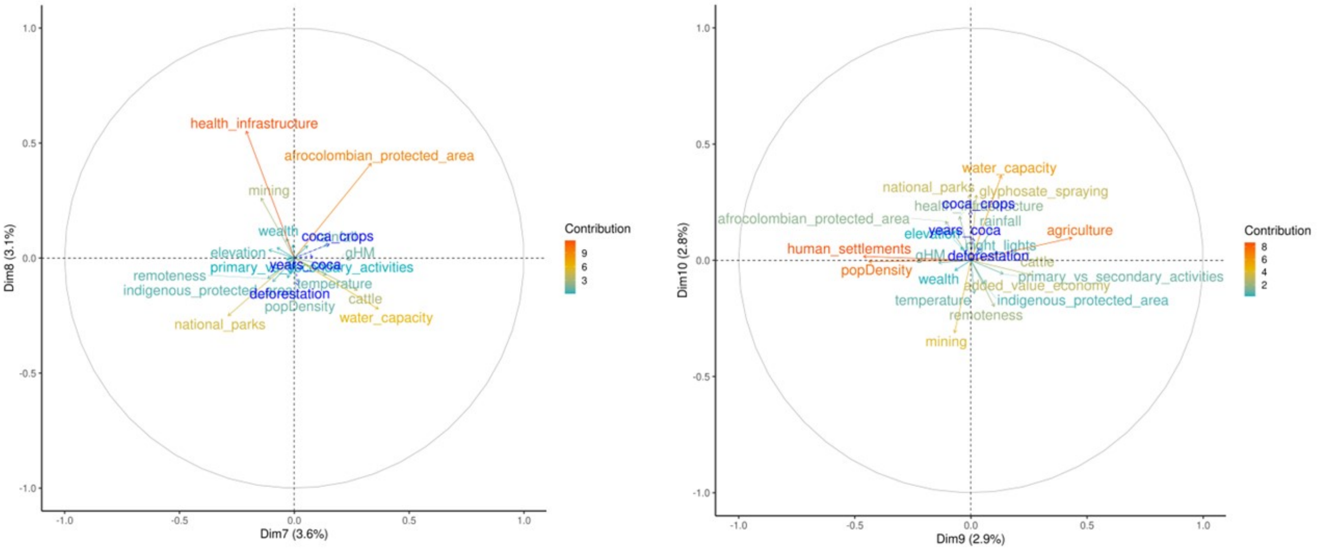

Figure S6. Variable contribution to dimension (left) 7 and 8 and (right) 9 and 10.

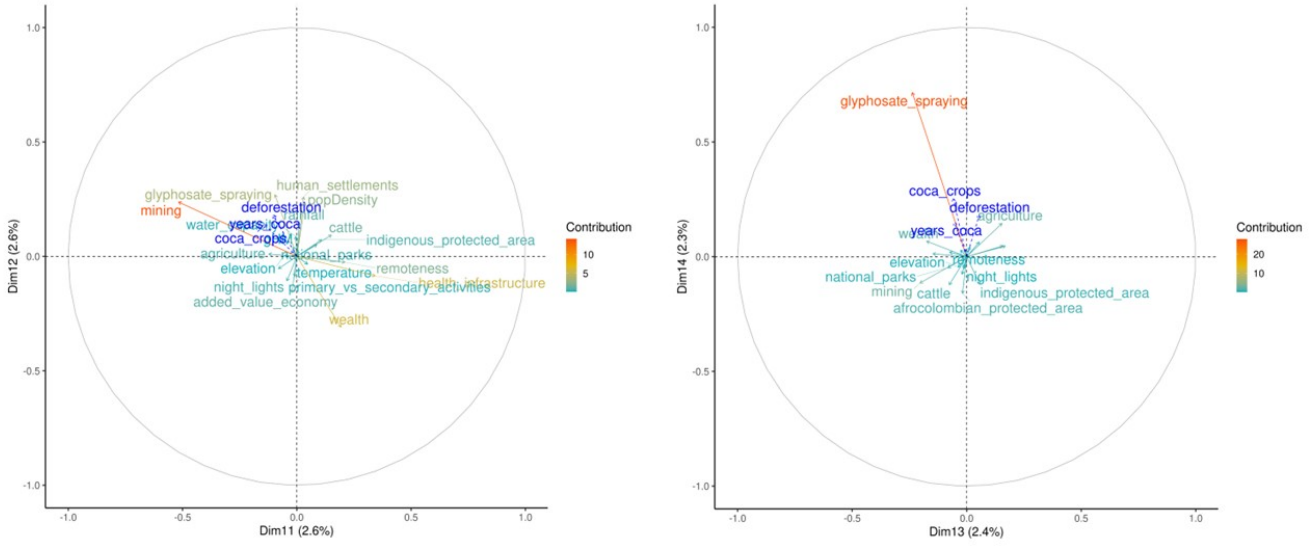

Figure S7. Variable contribution to dimension (left) 11 and 12 and (right) 13 and 14.

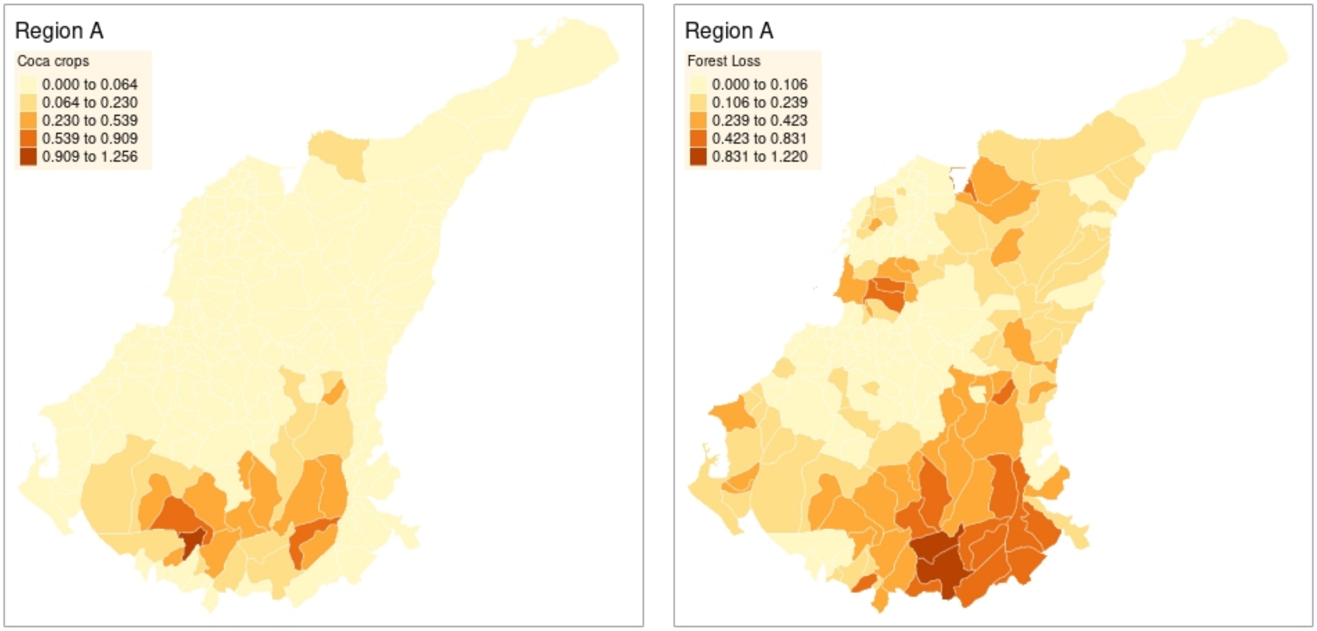

(a) **(b)**

Figure S8. Region A: (a) Map of average annual coca cultivation and (b) deforestation area, as a percentage of municipal area, between 2000 and 2020.

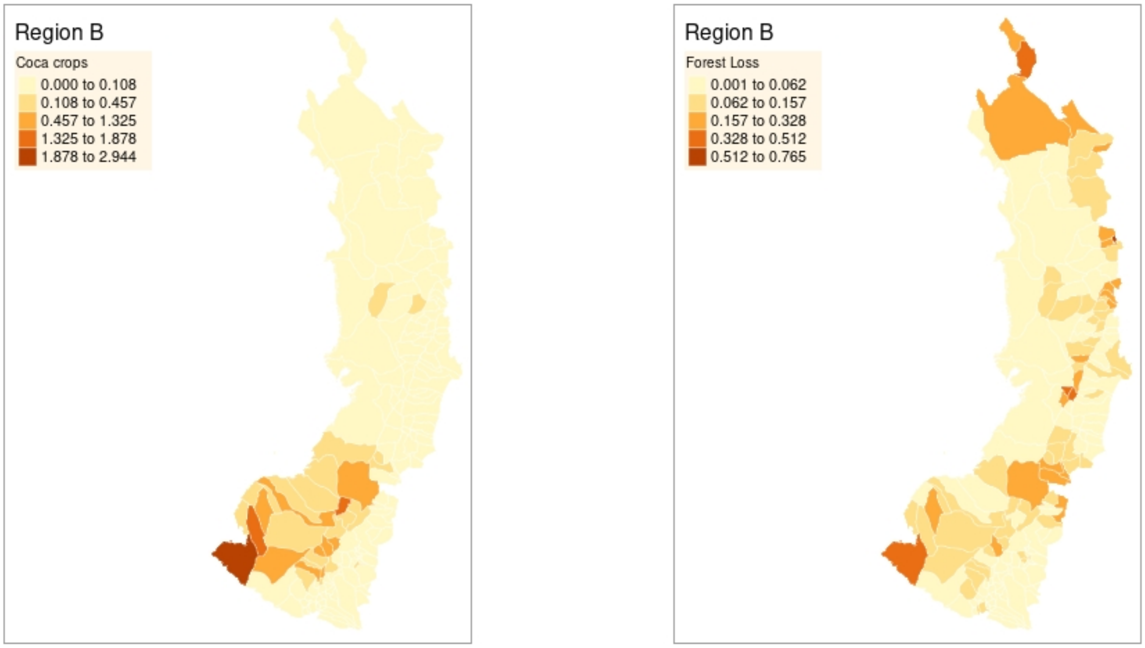

(a) **(b)**

Figure S9. Region B: (a) Map of average annual coca cultivation and (b) deforestation area, as percentage of municipal area, between 2000 and 2020.

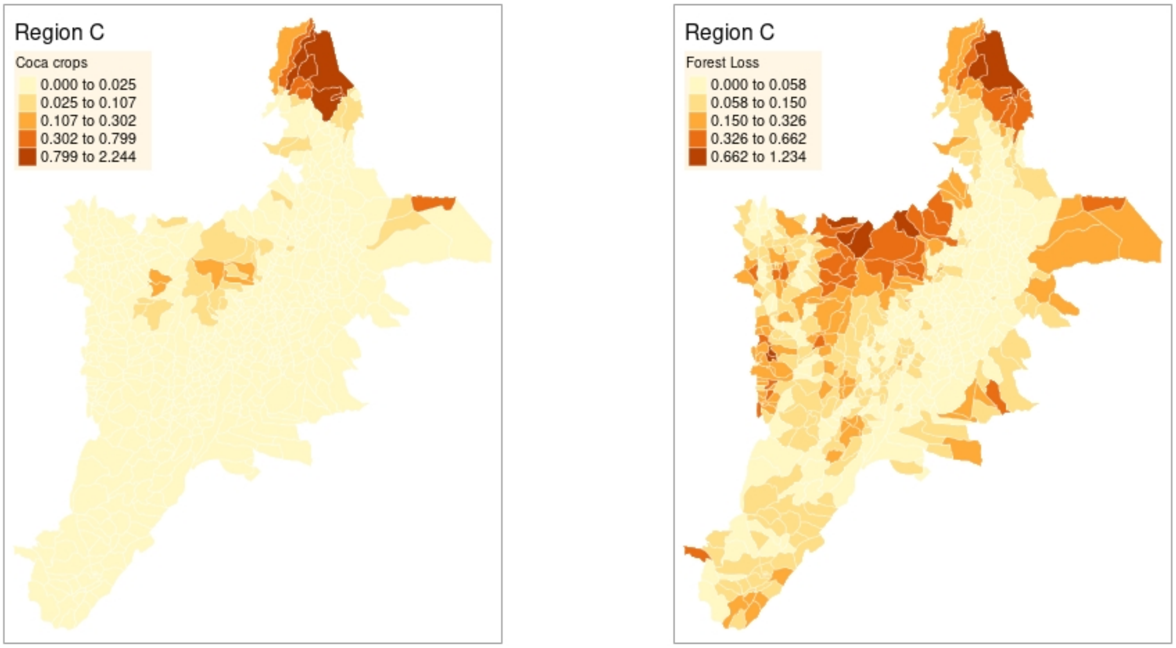

(a) **(b)**

Figure S10. Region C: (a) Map of average annual coca cultivation and (b) deforestation area, as percentage of municipal area, between 2000 and 2020.

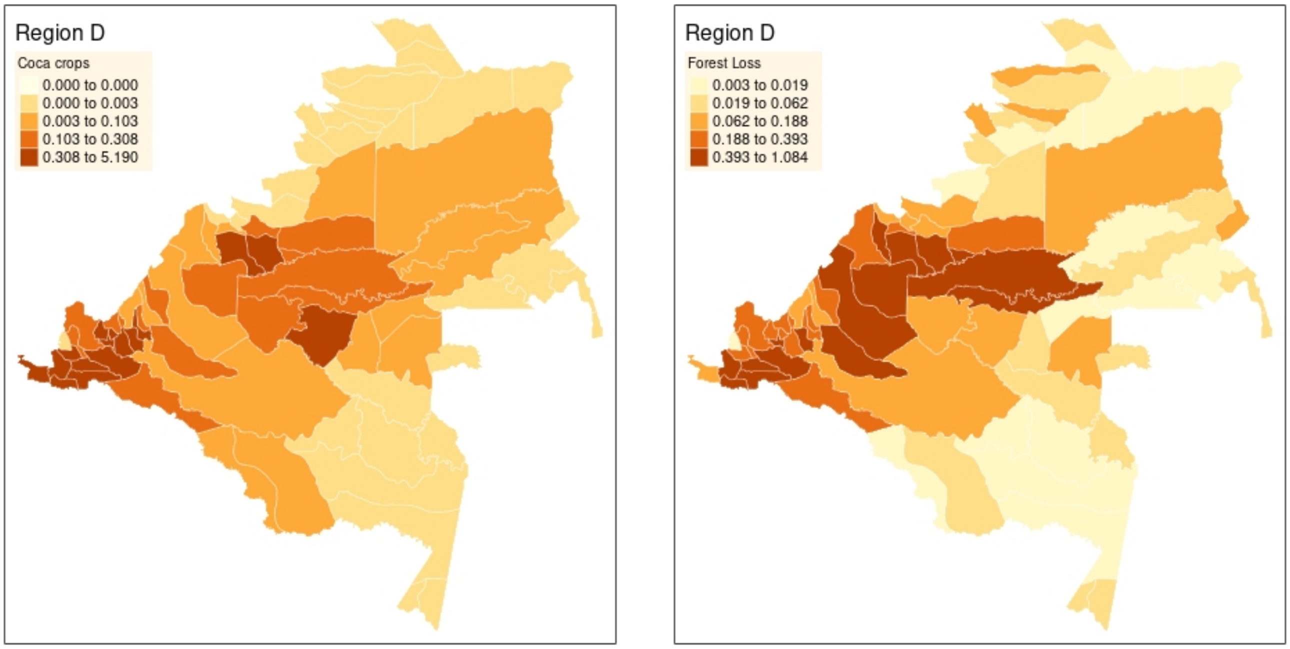

(a) **(b)**

Figure S11. Region D: (a) Map of average annual coca cultivation and (b) deforestation area, as percentage of municipal area, between 2000 and 2020.

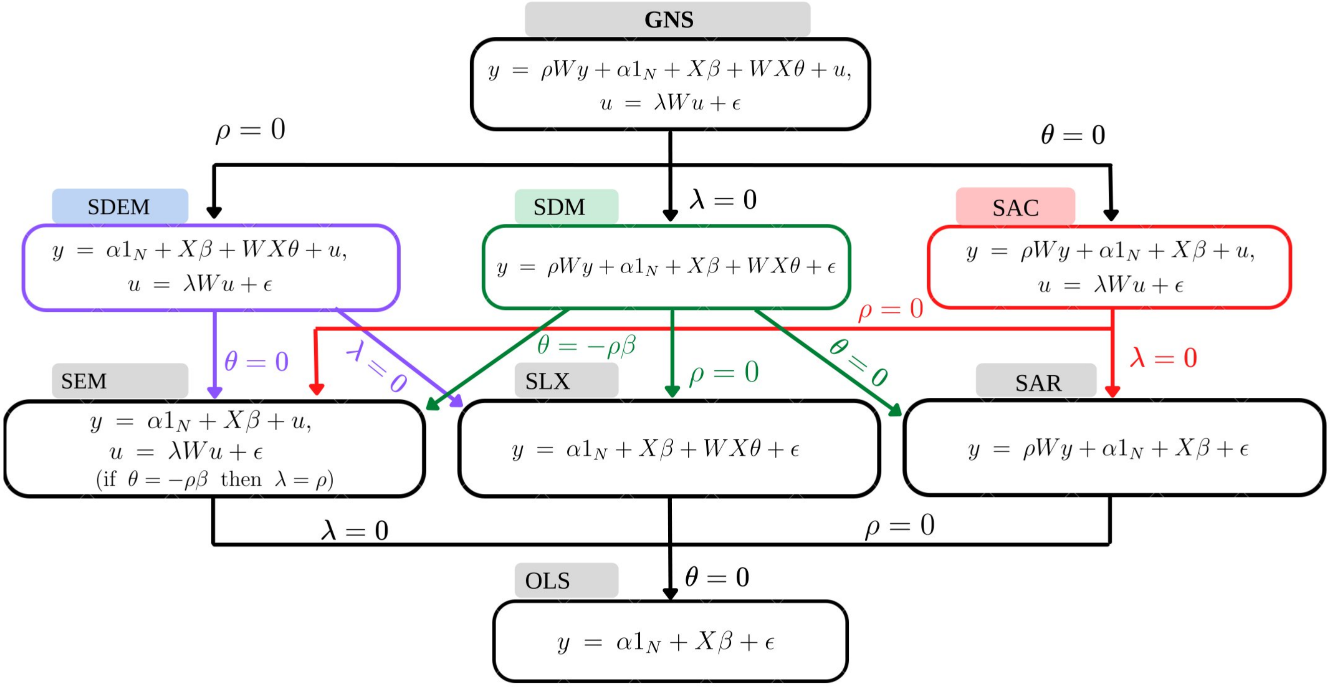

Figure S12. A Taxonomy of Linear Spatial Models. *Modified from*: [**?**]. OLS is ordinary least squares model, SEM is spatial error model, SLX is the spatial lag of X model, SAR is the spatial autoregressive model, SDEM is the Durbin error model, SDM is the Durbin model, SAC is the Spatial Autoregressive Combined Model and GNS is the General nesting model.

Table S2. Final Spatial Regression Results at the National and Regional Level.

		Regions			
	National	A	B	C	D
	SDEM	SLX	SLX	SDEM	SLX
(Intercept)	0.116	0.016	0.122	0.220	−0.063
	(0.334)	(0.048)	(0.126)	(0.681)	(0.079)
Coca crops	0.164***	−0.049	0.249**	0.303***	−0.063
	(0.028)	(0.063)	(0.081)	(0.039)	(0.11)
PC1	−0.317***	0.252	−0.3	0.365***	1.169***
	(0.052)	(0.139)	(0.215)	(0.074)	(0.226)
PC2	0.050*	−0.060	0.203*	−0.180***	—
	(0.026)	(0.074)	(0.09)	(0.045)	—
PC3	−0.336***	−0.238*	−0.192	−0.255***	—
	(0.037)	(0.092)	(0.122)	(0.049)	—
PC4	−0.040	0.133	−0.236*	−0.057	—
	(0.027)	(0.094)	(0.094)	(0.043)	—
PC5	−0.202***	−0.131	−0.12	−0.196**	−0.655**
	(0.038)	(0.112)	(0.131)	(0.061)	(0.232)
PC6	−0.157***	0.145	−0.43***	0.016	—
	(0.039)	(0.119)	(0.125)	(0.060)	—
PC7	−0.037	−0.113	0.407***	0.036	0.573***
	(0.031)	(0.093)	(0.114)	(0.047)	(0.129)
PC8	−0.065*	0.081	−0.233*	−0.038	—
	(0.027)	(0.066)	(0.111)	(0.047)	—
PC9	0.033	−0.157*	−0.006	−0.086*	−0.441**
	(0.025)	(0.07)	(0.125)	(0.034)	(0.152)
PC10	0.007	0.104	−0.009	0.016	—
	(0.029)	(0.084)	(0.096)	(0.044)	—
PC11	−0.073**	−0.162*	0.175	−0.109**	—
	(0.027)	(0.081)	(0.109)	(0.038)	—
PC12	−0.069**	−0.063	−0.095	−0.004	0.371*
	(0.026)	(0.067)	(0.13)	(0.038)	(0.147)
PC13	−0.096***	0.102	0.226**	0.137***	−0.433**
	(0.024)	(0.06)	(0.086)	(0.037)	(0.147)
PC14	0.114***	0.361***	−0.203	0.031	—
	(0.025)	(0.084)	(0.129)	(0.042)	—
**W** · **u**	0.932***	—	—	0.959***	—
	(0.028)	—	—	(0.027)	—
**W**·Coca crops	0.784**	0.466**	2.372*	−0.158	−0.358
	(0.253)	(0.146)	(0.931)	(0.368)	(0.376)
**W**·PC1	0.583**	0.171	−2.266*	1.063	−1.872**
	(0.198)	(0.212)	(0.076)	(0.909)	(0.676)
**W**·PC2	−0.070	−0.173	2.543*	−3.780***	—
	(0.337)	(0.155)	(1.077)	(0.909)	—
**W**·PC3	−0.042	0.121	0.877	−0.156	—
	(0.226)	(0.143)	(0.826)	(0.462)	—
**W**·PC4	−0.396	0.27	1.672	−4.312***	—
	(0.253)	(0.149)	(1.332)	(0.927)	—
**W**·PC5	−0.297	0.203	4.534***	0.268	0.859
	(0.189)	(0.192)	(1.343)	(0.813)	(0.533)
**W**·PC6	0.501**	0.036	−1.434	0.016	—
	(0.161)	(0.181)	(0.736)	(0.441)	—
**W**·PC7	−0.354	0.044	−0.967	−1.577**	−0.015
	(0.194)	(0.168)	(1.173)	(0.608)	(0.379)
**W**·PC8	−0.229	0.447***	−2.592*	−0.528	—
	(0.215)	(0.133)	(0.146)	(0.615)	—
**W**·PC9	0.005	−0.22	4.71*	−0.208	−0.294

	(0.261)	(0.189)	(1.892)	(0.526)	(0.427)
**W**·PC10	−0.251	0.181	−0.135	1.331**	—
	(0.180)	(0.139)	(1.065)	(0.438)	—
**W**·PC11	0.463	0.055	−3.071*	−0.893	—
	(0.242)	(0.152)	(1.293)	(0.515)	—
**W**·PC12	−0.625***	−0.087	−5.091**	−1.771***	−0.632
	(0.188)	(0.138)	(1.671)	(0.518)	(0.442)
**W**·PC13	0.107	0.12	4.141***	−1.063*	−0.489
	(0.232)	(0.13)	(1.043)	(0.494)	(0.489)
**W**·PC14	−0.173	0.407	2.154	0.219	—
	(0.137)	(0.232)	(1.871)	(0.545)	—
Adj. R^2	—	0.642	0.477	—	0.551
Num. obs.	1,060	196	173	613	78
Parameters	33	—	—	33	—
σ^2	0.517	—	—	0.454	—
Pseudo R^2	0.468	—	—	0.537	—
Wald test	1077.845***	—	—	1247.482***	—
Log.Lik(Linear)	−1207.901	—	—	−651.028	—
Log.Lik(Spatial)	−1168.683	—	—	−633.612	—
AIC (Linear)	2479.803	416.393	464.376	1366.056	187.16
AIC (Spatial)	2403.366	355.992	408.687	1333.223	174.29
LR test	78.437***	—	—	34.833***	—

Significance: $^{***}p \leq 0.001$; $^{**}p \leq 0.01$; $^{*}p \leq 0.05$

Table S3. Spatial Regression Results National Models

	Dependent variable: deforestation							
Independent variable	OLS	SLX	SAR	SEM	SAC	SDM	SDEM	GNS
(Intercept)	0.000	0.005	0.047*	0.065	−0.014	0.014	0.116	0.059
	(0.025)	(0.03)	(0.023)	(0.476)	(0.279)	(0.028)	(0.334)	(0.239)
Coca crops	0.226***	0.156***	0.224***	0.186***	0.183***	0.163***	0.164***	0.168***
	(0.029)	(0.03)	(0.027)	(0.028)	(0.027)	(0.028)	(0.028)	(0.029)
PC1	−0.200***	−0.301***	−0.123***	−0.294***	−0.245***	−0.299***	−0.317***	−0.326***
	(0.026)	(0.051)	(0.026)	(0.040)	(0.040)	(0.049)	(0.052)	(0.052)
PC2	0.012	0.047	−0.006	0.049	0.047	0.046	0.050*	0.048
	(0.025)	(0.027)	(0.024)	(0.025)	(0.024)	(0.025)	(0.026)	(0.025)
PC3	−0.252***	−0.335***	−0.177***	−0.351***	−0.314***	−0.329***	−0.336***	−0.329***
	(0.025)	(0.037)	(0.026)	(0.035)	(0.034)	(0.036)	(0.037)	(0.037)
PC4	−0.064*	−0.026	−0.050*	−0.038	−0.024	−0.029	−0.040	−0.041
	(0.025)	(0.028)	(0.023)	(0.026)	(0.025)	(0.027)	(0.027)	(0.027)
PC5	−0.288***	−0.218***	−0.192***	−0.240***	−0.217***	−0.218***	−0.202***	−0.196***
	(0.025)	(0.039)	(0.024)	(0.034)	(0.033)	(0.037)	(0.038)	(0.037)
PC6	0.072**	−0.144***	0.014	−0.090**	−0.090**	−0.124***	−0.157***	−0.145***
	(0.025)	(0.039)	(0.023)	(0.035)	(0.033)	(0.037)	(0.039)	(0.038)
PC7	−0.020	−0.055	−0.040	−0.037	−0.028	−0.040	−0.037	−0.032
	(0.025)	(0.031)	(0.024)	(0.030)	(0.028)	(0.030)	(0.031)	(0.031)
PC8	−0.132***	−0.069	−0.075**	−0.104***	−0.082**	−0.068*	−0.065*	−0.064*
	(0.025)	(0.028)*	(0.023)	(0.026)	(0.025)	(0.027)	(0.027)	(0.027)
PC9	0.042	0.038	0.057*	0.034	0.032	0.036	0.033	0.035
	(0.025)	(0.026)	(0.023)	(0.025)	(0.024)	(0.025)	(0.025)	(0.025)
PC10	−0.008	0.012	−0.014	0.001	−0.010	0.009	0.007	0.009
	(0.026)	(0.03)	(0.024)	(0.028)	(0.027)	(0.028)	(0.029)	(0.049)
PC11	−0.070**	−0.083**	−0.041	−0.056*	−0.054*	−0.073**	−0.073**	−0.065*
	(0.025)	(0.028)	(0.024)	(0.026)	(0.025)	(0.027)	(0.027)	(0.026)

PC12	−0.150***	−0.079**	−0.099***	−0.086***	−0.069**	−0.073**	−0.069**	−0.063*
	(0.025)	(0.027)	(0.024)	(0.026)	(0.025)	(0.026)	(0.026)	(0.025)
PC13	−0.069**	−0.097***	−0.091***	−0.081***	−0.086***	−0.094***	−0.096***	−0.094***
	(0.025)	(0.026)	(0.023)	(0.024)	(0.023)	(0.024)	(0.024)	(0.025)
PC14	0.124***	0.121***	0.094***	0.113***	0.102***	0.113***	0.114***	0.105***
	(0.026)	(0.027)	(0.024)	(0.025)	(0.025)	(0.025)	(0.025)	(0.025)
**W** $\cdot y$	—	—	0.827***	—	0.791***	0.877***	—	0.791***
	—	—	(0.042)	—	(0.161)	(0.042)	—	(0.088)
**W** $\cdot u$	—	—	—	0.952***	0.893***	—	0.932***	0.893***
	—	—	—	(0.022)	(0.106)	—	(0.028)	(0.070)
**W**·coca_crops	—	1.058***	—	—	—	0.584***	0.784**	0.438
	—	(0.175)	—	—	—	(0.167)	(0.253)	(0.245)
**W**·PC1	—	0.376***	—	—	—	0.487***	0.583**	0.672**
	—	(0.119)	—	—	—	(0.112)	(0.198)	(0.209)
**W**·PC2	—	0.0329	—	—	—	−0.024	−0.070	−0.028
	—	(0.269)	—	—	—	(0.254)	(0.337)	(0.703)
**W**·PC3	—	0.04	—	—	—	0.226*	−0.042	0.136
	—	(0.098)	—	—	—	(0.093)	(0.226)	(0.217)
**W**·PC4	—	−0.105	—	—	—	−0.071	−0.396	−0.290
	—	(0.185)	—	—	—	(0.175)	(0.253)	(0.329)
**W**·PC5	—	−0.089	—	—	—	0.133	−0.297	−0.082
	—	(0.117)	—	—	—	(0.112)	(0.189)	(0.246)
**W**·PC6	—	0.371***	—	—	—	0.161	0.501**	0.300
	—	(0.094)	—	—	—	(0.090)	(0.161)	(0.173)
**W**·PC7	—	−0.187	—	—	—	−0.081	−0.354	−0.232
	—	(0.105)	—	—	—	(0.100)	(0.194)	(0.193)
**W**·PC8	—	−0.398**	—	—	—	−0.198	−0.229	−0.102
	—	(0.166)	—	—	—	(0.157)	(0.215)	(0.223)
**W**·PC9	—	−0.143	—	—	—	−0.135	0.005	−0.038
	—	(0.179)	—	—	—	(0.169)	(0.261)	(0.291)
**W**·PC10	—	−0.261*	—	—	—	−0.146	−0.251	−0.138
	—	(0.127)	—	—	—	(0.120)	(0.180)	(0.205)
**W**·PC11	—	0.598**	—	—	—	0.512**	0.463	0.477
	—	(0.183)	—	—	—	(0.173)	(0.242)	(0.260)
**W**·PC12	—	−0.478***	—	—	—	−0.217	−0.625***	−0.359
	—	(0.123)	—	—	—	(0.117)	(0.188)	(0.195)
**W**·PC13	—	0.164	—	—	—	0.053	0.107	0.062
	—	(0.201)	—	—	—	(0.190)	(0.232)	(0.247)
**W**·PC14	—	−0.154	—	—	—	−0.259*	−0.173	−0.224
	—	(0.114)	—	—	—	(0.108)	(0.137)	(0.141)
R^2	0.358	0.428	—	—	—	—	—	—
Adj. R^2	0.348	0.411	—	—	—	—	—	—
Num. obs.	1,060	1,060	1,060	1,060	1,060	1,060	1,060	1,060
Parameters	—	—	18	18	19	33	33	34
σ^2	—	—	0.573	0.542	0.508	0.524	0.517	0.493
Pseudo R^2	—	—	0.416	0.441	0.470	0.464	0.468	0.487
Wald test	—	—	393.533***	1832.173***	225.941***	426.847***	1077.845***	162.098***
Log.Lik(Linear)	—	—	−1268.975	−1268.975	−1268.975	−1207.901	−1207.901	−1268.975
Log.Lik(Spatial)	—	—	−1218.146	−1195.510	−1166.956	−1173.019	−1168.683	−1149.712
AIC (Linear)	2571.950	2571.950	2571.950	2571.950	2571.950	2479.803	2479.803	2571.950
AIC (Spatial)	—	2479.803}	2472.291	2427.020	2371.912	2412.038	2403.366	2367.424
LR test	—	—	101.659***	146.930***	204.038***	69.765***	78.437***	238.526***

*** $p < 0.001$; ** $p < 0.01$; * $p < 0.05$

Table S4. Spatial Regression Results Region A

Dependent variable: deforestation

Independent variable	OLS	SLX	SAR	SEM	SAC	SDM	SDEM	GNS
(Intercept)	−0.000	0.016	0.182***	0.069	0.234	0.139	0.170	0.132
	(0.048)	(0.048)	(0.043)	(0.091)	(0.132)	(0.386)	(0.392)	(0.412)
Coca crops	0.121*	−0.049	0.090	0.057	0.035	−0.009	−0.008	−0.009
	(0.059)	(0.063)	(0.054)	(0.055)	(0.053)	(0.049)	(0.050)	(0.053)
PC1	0.293**	0.252	0.246**	0.312***	0.266	0.147	0.144	0.149
	(0.094)	(0.139)	(0.085)	(0.090)	(0.087)	(0.106)	(0.106)	(0.107)
PC2	−0.035	−0.060	−0.068	−0.018	−0.035	−0.066	−0.067	−0.067
	(0.069)	(0.074)	(0.063)	(0.064)	(0.063)	(0.062)	(0.062)	(0.062)
PC3	−0.138*	−0.238*	−0.199***	−0.191**	−0.230	−0.114	−0.115	−0.116
	(0.062)	(0.092)	(0.056)	(0.064)	(0.064)	(0.067)	(0.068)	(0.068)
PC4	0.194*	0.133	0.099	0.170*	0.099	0.080	0.079	0.079
	(0.083)	(0.094)	(0.075)	(0.078)	(0.078)	(0.088)	(0.088)	(0.089)
PC5	−0.144	−0.131	−0.053	−0.211*	−0.156	−0.118	−0.120	−0.118
	(0.087)	(0.112)	(0.081)	(0.085)	(0.084)	(0.087)	(0.087)	(0.087)
PC6	0.014	0.145	0.154*	0.008	0.105	0.198*	0.200*	0.200*
	(0.075)	(0.119)	(0.069)	(0.077)	(0.076)	(0.092)	(0.092)	(0.092)
PC7	−0.098	−0.113	−0.054	−0.117	−0.087	−0.138	−0.139	−0.136
	(0.076)	(0.093)	(0.069)	(0.073)	(0.071)	(0.072)	(0.072)	(0.072)
PC8	0.210**	0.081	0.193**	0.128*	0.106	0.145*	0.147*	0.144*
	(0.065)	(0.066)	(0.060)	(0.061)	(0.06)	(0.059)	(0.059)	(0.058)
PC9	−0.174*	−0.157*	−0.108	−0.197**	−0.150	−0.200*	−0.197*	−0.197*
	(0.071)	(0.07)	(0.064)	(0.062)	(0.064)	(0.078)	(0.079)	(0.079)
PC10	0.250***	0.104	0.241***	0.216***	0.191	0.252***	0.251***	0.249***
	(0.060)	(0.084)	(0.055)	(0.062)	(0.058)	(0.066)	(0.066)	(0.066)
PC11	−0.262***	−0.162*	−0.144*	−0.264***	−0.179	−0.197**	−0.194**	−0.193**
	(0.064)	(0.081)	(0.059)	(0.064)	(0.066)	(0.068)	(0.068)	(0.069)
PC12	−0.121*	−0.063	−0.083	−0.132*	−0.111	−0.038	−0.039	−0.039
	(0.058)	(0.067)	(0.053)	(0.055)	(0.05)	(0.054)	(0.055)	(0.054)
PC13	0.130*	0.102	0.083	0.124*	0.098	0.101*	0.104*	0.101*
	(0.056)	(0.06)	(0.051)	(0.052)	(0.05)	(0.049)	(0.049)	(0.049)
PC14	0.478***	0.361***	0.467***	0.457***	0.444	0.457***	0.457***	0.456***
	(0.086)	(0.084)	(0.077)	(0.076)	(0.072)	(0.072)	(0.072)	
$\mathbf{W} \cdot y$	—	—	0.969***	—	0.964	0.311	—	0.292
	—	—	(0.069)	—	(0.59)	(0.207)	—	(0.291)
$\mathbf{W} \cdot u$	—	—	—	0.999***	0.999	—	0.213	0.146
	—	—	—	(0.061)	(0.61)	—	(0.249)	(0.394)
**W**·coca_crops	—	0.466**	—	—	—	1.708	1.884*	1.666
	—	(0.146)	—	—	—	(0.944)	(0.947)	(0.995)
**W**·PC1	—	0.171	—	—	—	1.677	1.739	1.714
	—	(0.212)	—	—	—	(1.053)	(1.063)	(1.053)
**W**·PC2	—	−0.173	—	—	—	−1.918	−1.933	−1.919
	—	(0.155)	—	—	—	(1.031)	(1.044)	(1.045)
**W**·PC3	—	0.121	—	—	—	−0.620	−0.680	−0.588
	—	(0.143)	—	—	—	(0.504)	(0.509)	(0.527)
**W**·PC4	—	0.27	—	—	—	1.620	1.696	1.653
	—	(0.149)	—	—	—	(0.949)	(0.960)	(0.962)
**W**·PC5	—	0.203	—	—	—	1.657	1.614	1.653
	—	(0.192)	—	—	—	(0.866)	(0.879)	(0.869)
**W**·PC6	—	0.036	—	—	—	1.841*	1.880**	1.832*
	—	(0.181)	—	—	—	(0.717)	(0.730)	(0.730)

**W**·PC7	—	0.044	—	—	—	0.145	0.130	0.150
	—	(0.168)	—	—	—	(0.833)	(0.843)	(0.838)
**W**·PC8	—	0.447***	—	—	—	2.899**	3.007***	2.914**
	—	(0.133)	—	—	—	(0.895)	(0.904)	(0.902)
**W**·PC9	—	−0.22	—	—	—	−1.788*	−1.802*	−1.781*
	—	(0.189)	—	—	—	(0.872)	(0.876)	(0.875)
**W**·PC10	—	0.181	—	—	—	0.323	0.390	0.365
	—	(0.139)	—	—	—	(0.548)	(0.555)	(0.565)
**W**·PC11	—	0.055	—	—	—	0.631	0.607	0.626
	—	(0.152)	—	—	—	(0.712)	(0.719)	(0.717)
**W**·PC12	—	−0.087	—	—	—	−1.549*	−1.603*	−1.570*
	—	(0.138)	—	—	—	(0.687)	(0.700)	(0.698)
**W**·PC13	—	0.12	—	—	—	0.237	0.263	0.229
	—	(0.13)	—	—	—	(0.650)	(0.665)	(0.660)
**W**·PC14	—	0.407	—	—	—	3.906**	4.086**	3.941**
	—	(0.232)	—	—	—	(1.298)	(1.304)	(1.335)
R^2	0.586	0.697	—	—	—	—	—	—
Adj. R^2	0.551	0.642	—	—	—	—	—	—
Num. obs.	196	196	196	196	196	196	196	196
Parameters	—	—	18	18	19	34	34	35
σ^2	—	—	0.367	0.354	0.321	0.255	0.256	0.255
Pseudo R^2	—	—	0.624	0.637	0.663	0.743	0.742	0.743
Wald test	—	—	199.452***	264.933	12.82**	2.264	0.736	0.137
LogLik(Linear)	—	—	−191.197	−191.197	−191.197	−144.996	−144.996	−191.197
LogLik(Spatial)	—	—	−181.806	−178.398	−170.986	−144.513	−144.845	−144.451
AIC (Linear)	416.393	416.393	416.393	416.393	416.393	355.992	355.992	416.393
AIC (Spatial)	—	355.992	399.613	392.796	379.973	357.026	357.690	358.902
LR test	—	—	18.781***	25.598***	40.421***	0.967	0.302	93.492***

*** $p < 0.001$; ** $p < 0.01$; * $p < 0.05$

Table S5. Spatial Regression Results Region B

	Dependent variable: deforestation							
Independent variable	OLS	SLX	SAR	SEM	SAC	SDM	SDEM	GNS
(Intercept)	0.000	0.122	0.023	0.210	0.074	0.113	0.112	0.112
	(0.067)	(0.126)	(0.061)	(0.391)	(0.189)	(0.113)	(0.129)	(0.112)
Coca crops	0.235**	0.249**	0.263***	0.238**	0.240**	0.249***	0.249***	0.249***
	(0.084)	(0.081)	(0.075)	(0.082)	(0.079)	(0.072)	(0.072)	(0.071)
PC1	0.198	−0.3	0.071	0.042	0.049	−0.278	−0.288	−0.277
	(0.144)	(0.215)	(0.129)	(0.160)	(0.152)	(0.192)	(0.194)	(0.154)
PC2	0.150	0.203*	0.139	0.128	0.119	0.195*	0.201*	0.195*
	(0.086)	(0.09)	(0.077)	(0.084)	(0.081)	(0.081)	(0.082)	(0.077)
PC3	−0.395***	−0.192	−0.299**	−0.363**	−0.342**	−0.201	−0.194	−0.201
	(0.108)	(0.122)	(0.098)	(0.117)	(0.111)	(0.109)	(0.109)	(0.106)
PC4	−0.103	−0.236*	−0.143	−0.174*	−0.173*	−0.233**	−0.230**	−0.232**
	(0.093)	(0.094)	(0.083)	(0.087)	(0.085)	(0.084)	(0.085)	(0.079)
PC5	−0.355**	−0.12	−0.290**	−0.369**	−0.346**	−0.122	−0.117	−0.121
	(0.122)	(0.131)	(0.110)	(0.120)	(0.116)	(0.117)	(0.118)	(0.117)
PC6	−0.266*	−0.43***	−0.179	−0.247*	−0.207	−0.396***	−0.408***	−0.394***
	(0.110)	(0.125)	(0.100)	(0.117)	(0.112)	(0.112)	(0.114)	(0.110)

PC7	0.271*	0.407***	0.285**	0.307**	0.308**	0.390***	0.399***	0.389***
	(0.123)	(0.114)	(0.110)	(0.112)	(0.111)	(0.102)	(0.103)	(0.085)
PC8	−0.324**	−0.233*	−0.266*	−0.272*	−0.264*	−0.210*	−0.227*	−0.210*
	(0.121)	(0.111)	(0.109)	(0.111)	(0.109)	(0.099)	(0.100)	(0.085)
PC9	−0.155	−0.006	−0.168	−0.188	−0.195	−0.015	−0.002	−0.014
	(0.127)	(0.125)	(0.114)	(0.114)	(0.113)	(0.112)	(0.113)	(0.113)
PC10	−0.179	−0.009	−0.138	−0.117	−0.111	−0.003	−0.005	−0.002
	(0.097)	(0.096)	(0.087)	(0.091)	(0.089)	(0.086)	(0.087)	(0.086)
PC11	0.127	0.175	0.137	0.169	0.167	0.170	0.166	0.168
	(0.107)	(0.109)	(0.095)	(0.101)	(0.099)	(0.097)	(0.098)	(0.097)
PC12	−0.061	−0.095	0.016	0.030	0.049	−0.079	−0.095	−0.080
	(0.125)	(0.13)	(0.112)	(0.115)	(0.114)	(0.116)	(0.118)	(0.118)
PC13	0.255**	0.226**	0.190*	0.214*	0.194*	0.218**	0.226**	0.218***
	(0.086)	(0.086)	(0.077)	(0.085)	(0.083)	(0.077)	(0.077)	(0.058)
PC14	−0.132	−0.203	−0.156	−0.194	−0.186	−0.189	−0.191	−0.187*
	(0.135)	(0.129)	(0.121)	(0.126)	(0.124)	(0.115)	(0.116)	(0.088)
$\mathbf{W} \cdot y$	—	—	0.814***	—	0.653**	0.531**	—	0.517*
	—	—	(0.093)	—	(0.215)	(0.194)	—	(0.258)
$\mathbf{W} \cdot u$	—	—	—	0.845***	0.665**	—	0.363	0.066
	—	—	—	(0.083)	(0.222)	—	(0.250)	(0.94)
**W**·coca_crops	—	2.372*	—	—	—	2.287**	2.349**	2.288**
	—	(0.931)	—	—	—	(0.832)	(0.861)	(0.823)
**W**·PC1	—	−2.266*	—	—	—	−2.572**	−2.350**	−2.587***
	—	(0.076)	—	—	—	(0.786)	(0.805)	(0.655)
**W**·PC2	—	2.543*	—	—	—	2.546**	2.623**	2.566
	—	(1.077)	—	—	—	(0.962)	(0.983)	(0.97)
**W**·PC3	—	0.877	—	—	—	1.178	0.880	1.174
	—	(0.826)	—	—	—	(0.745)	(0.771)	(0.748)
**W**·PC4	—	1.672	—	—	—	1.695	1.691	1.699
	—	(1.332)	—	—	—	(1.190)	(1.213)	(1.138)
**W**·PC5	—	4.534***	—	—	—	4.798***	4.563***	4.810***
	—	(1.343)	—	—	—	(1.200)	(1.212)	(0.630)
**W**·PC6	—	−1.434	—	—	—	−1.374*	−1.451*	−1.379*
	—	(0.736)	—	—	—	(0.657)	(0.695)	(0.653)
**W**·PC7	—	−0.967	—	—	—	−1.503	−1.116	−1.523***
	—	(1.173)	—	—	—	(1.055)	(1.083)	(0.274)
**W**·PC8	—	−2.592*	—	—	—	−1.976	−2.417*	−1.957**
	—	(0.146)	—	—	—	(1.036)	(1.071)	(0.654)
**W**·PC9	—	4.71*	—	—	—	4.794**	4.812**	4.825***
	—	(1.892)	—	—	—	(1.689)	(1.712)	(0.161)
**W**·PC10	—	−0.135	—	—	—	0.252	−0.064	0.260
	—	(1.065)	—	—	—	(0.956)	(0.972)	(1.005)
**W**·PC11	—	−3.071*	—	—	—	−3.137**	−3.093**	−3.150
	—	(1.293)	—	—	—	(1.154)	(1.170)	(1.15)
**W**·PC12	—	−5.091**	—	—	—	−5.130***	−5.109***	−5.147***
	—	(1.671)	—	—	—	(1.492)	(1.497)	(0.523)
**W**·PC13	—	4.141***	—	—	—	3.906***	4.010***	3.898***
	—	(1.043)	—	—	—	(0.933)	(0.954)	(0.868)
**W**·PC14	—	2.154	—	—	—	2.520	2.268	2.543**
	—	(1.871)	—	—	—	(1.672)	(1.700)	(0.978)
R^2	0.291	0.568	—	—	—	—	—	—
Adj. R^2	0.224	0.477	—	—	—	—	—	—
Num. obs.	173	173	173	173	173	173	173	173
Parameters	—	—	18	18	19	33	33	34
σ^2	—	—	0.621	0.621	0.602	0.417	0.426	0.417

Pseudo R^2	—	—	0.356	0.354	0.375	0.577	0.570	0.577
Wald test	—	—	76.479***	102.800***	8.943*	7.454**	2.105	0.005
Log.Lik(Linear)	—	—	−215.188	−215.188	−215.188	−172.343	−172.343	−215.188
Log Lik(Spatial)	—	—	−206.916	−207.164	−204.338	−170.619	−171.932	−170.611
AIC (Linear)	464.376	464.376	464.376	464.376	464.376	408.687	408.687	464.376
AIC (Spatial)	—	408.687	449.832	450.329	446.677	407.239	409.863	409.221
LR test	—	—	16.543***	16.047***	21.699***	3.448	0.823	89.155***

***$p < 0.001$; **$p < 0.01$; *$p < 0.05$

Table S6. Spatial Regression Results Region C

	Dependent variable: deforestation							
Independent variable	OLS	SLX	SAR	SEM	SAC	SDM	SDEM	GNS
(Intercept)	0.000	−0.008	0.050	0.285	0.079	−0.003	0.220	0.126
	(0.031)	(0.048)	(0.029)	(0.682)	(0.660)	(0.045)	(0.681)	(0.319)
Coca crops	0.324***	0.315***	0.265***	0.321***	0.289***	0.309***	0.303***	0.299***
	(0.034)	(0.041)	(0.033)	(0.036)	(0.035)	(0.039)	(0.039)	(0.038)
PC1	0.249***	0.328***	0.224***	0.265***	0.231***	0.326***	0.365***	0.355***
	(0.061)	(0.077)	(0.058)	(0.061)	(0.060)	(0.073)	(0.074)	(0.070)
PC2	−0.210***	−0.197***	−0.165***	−0.181***	−0.167***	−0.183***	−0.180***	−0.167***
	(0.047)	(0.048)	(0.045)	(0.045)	(0.044)	(0.045)	(0.045)	(0.044)
PC3	−0.204***	−0.277***	−0.172***	−0.221***	−0.210***	−0.267***	−0.255***	−0.250
	(0.042)	(0.051)	(0.040)	(0.044)	(0.042)	(0.048)	(0.049)	0.048
PC4	−0.079	−0.073	−0.057	−0.045	−0.046	−0.061	−0.057	−0.049
	(0.045)	(0.046)	(0.043)	(0.043)	(0.042)	(0.043)	(0.043)	(0.040)
PC5	−0.152**	−0.193**	−0.117*	−0.148*	−0.155**	−0.201**	−0.196**	−0.202***
	(0.058)	(0.065)	(0.055)	(0.058)	(0.056)	(0.062)	(0.061)	(0.058)
PC6	−0.122**	0.019	−0.010	−0.024	0.010	0.007	0.016	0.008
	(0.042)	(0.063)	(0.041)	(0.049)	(0.045)	(0.059)	(0.060)	0.059
PC7	0.108*	0.038	0.065	0.082	0.067	0.046	0.036	0.042
	(0.045)	(0.049)	(0.043)	(0.045)	(0.044)	(0.047)	(0.047)	(0.026)
PC8	−0.066	−0.051	−0.035	−0.071	−0.060	−0.046	−0.038	−0.035
	(0.047)	(0.05)	(0.045)	(0.046)	(0.044)	(0.047)	(0.047)	(0.032)
PC9	−0.068*	−0.064	−0.097**	−0.082*	−0.082**	−0.071*	−0.086*	−0.089**
	(0.034)	(0.035)	(0.033)	(0.033)	(0.032)	(0.033)	(0.034)	(0.032)
PC10	0.059	0.005	0.051	0.040	0.042	0.012	0.016	0.017
	(0.038)	(0.046)	(0.036)	(0.038)	(0.037)	(0.044)	(0.044)	(0.031)
PC11	−0.158***	−0.108**	−0.125***	−0.125***	−0.116**	−0.110**	−0.109**	−0.109**
	(0.036)	(0.04)	(0.035)	(0.036)	(0.035)	(0.038)	(0.038)	(0.037)
PC12	−0.029	−0.006	−0.021	−0.013	0.003	−0.000	−0.004	0.000
	(0.036)	(0.04)	(0.04)	(0.035)	(0.036)	(0.042)	(0.038)	(0.038)
PC13	0.090*	0.132***	0.125***	0.118***	0.130***	0.133***	0.137***	0.136***
	(0.035)	(0.039)	(0.033)	(0.034)	(0.034)	(0.037)	(0.037)	(0.034)
PC14	0.030	0.029	0.043	0.038	0.042	0.032	0.031	0.033
	(0.043)	(0.045)	(0.041)	(0.041)	(0.040)	(0.042)	(0.042)	(0.034)
$\mathbf{W} \cdot y$	—	—	0.780***	—	0.877***	0.908***	—	0.870***
	—	—	(0.075)	—	(0.095)	(0.055)	—	(0.104)
$\mathbf{W} \cdot u$	—	—	—	0.958***	0.948***	—	0.959***	0.944***
	—	—	—	(0.028)	(0.048)	—	(0.027)	(0.058)

**W**·coca_crops	—	−0.084	—	—	—	−0.314	−0.158	−0.386
	—	0.291	—	—	—	(0.275)	(0.368)	(0.357)
**W**·PC1	—	0.706	—	—	—	0.292	1.063	0.699
	—	(0.839)	—	—	—	(0.793)	(0.909)	(0.864)
**W**·PC2	—	−3.239***	—	—	—	−2.598***	−3.780***	−3.162***
	—	(0.826)	—	—	—	(0.781)	(0.909)	(0.870)
**W**·PC3	—	−0.304	—	—	—	−0.176	−0.156	−0.021
	—	(0.413)	—	—	—	(0.390)	(0.462)	0.449
**W**·PC4	—	−5.16***	—	—	—	−3.766***	−4.312***	−3.713***
	—	(0.872)	—	—	—	(0.824)	(0.927)	(0.865)
**W**·PC5	—	−0.521	—	—	—	−0.249	0.268	0.417
	—	(0.754)	—	—	—	(0.712)	(0.813)	(0.693)
**W**·PC6	—	0.266	—	—	—	0.555	0.016	0.317
	—	(0.375)	—	—	—	(0.355)	(0.441)	0.468
**W**·PC7	—	−2.258***	—	—	—	−1.916***	−1.577**	−1.515***
	—	(0.538)	—	—	—	(0.508)	(0.608)	(0.333)
**W**·PC8	—	−0.548	—	—	—	−0.631	−0.528	−0.597
	—	(0.561)	—	—	—	(0.530)	(0.615)	(0.520)
**W**·PC9	—	−0.497	—	—	—	−0.346	−0.208	−0.077
	—	(0.499)	—	—	—	(0.471)	(0.526)	(0.375)
**W**·PC10	—	1.781***	—	—	—	1.359***	1.331**	1.062**
	—	(0.379)	—	—	—	(0.358)	(0.438)	(0.410)
**W**·PC11	—	−0.923	—	—	—	−0.594	−0.893	−0.603
	—	(0.486)	—	—	—	(0.459)	(0.515)	0.514
**W**·PC12	—	−2.053***	—	—	—	−1.867***	−1.771***	−1.646***
	—	(0.491)	—	—	—	(0.463)	(0.518)	(0.231)
**W**·PC13	—	−0.998*	—	—	—	−0.902*	−1.063*	−0.970*
	—	(0.457)	—	—	—	(0.431)	(0.494)	(0.474)
**W**·PC14	—	0.418	—	—	—	0.410	0.219	0.263
	—	(0.497)	—	—	—	(0.469)	(0.545)	(0.517)
R^2	0.441	0.809	—	—	—	—	—	
Adj. R^2	0.427	0.484	—	—	—	—	—	
Num. obs.	613	613	613	613	613	613	613	613
Parameters	—	—	18	18	19	33	33	34
σ^2	—	—	0.519	0.498	0.468	0.460	0.454	0.434
Pseudo R^2	—	—	0.476	0.492	0.517	0.533	0.537	0.552
Wald test	—	—	108.959***	1200.99***	387.523***	272.391***	1247.482***	264.525***
LogLik (Linear)	—	—	−691.110	−691.110	−691.110	−651.028	−651.028	−691.110
LogLik (Spatial)	—	—	−671.372	−661.861	−646.466	−635.954	−633.612	−622.988
AIC (Linear)	1416.219	1416.219	1416.219	1416.219	1416.219	1366.056	1366.056	1416.219
AIC (Spatial)	—	1366.06	1378.744	1359.723	1330.933	1337.908	1333.223	1313.977
LR test	—	—	39.476***	58.496***	89.287***	30.148***	34.833***	136.242***

*** $p < 0.001$; ** $p < 0.01$; * $p < 0.05$

Table S7. Spatial Regression Results Region D

Dependent variable: deforestation								
Independent variable	OLS	SLX	SAR	SEM	SAC	SDM	SDEM	GNS

(Intercept)	0.000	−0.063	0.000	−0.056	−0.012	−0.056	−0.076	−0.069
	(0.086)	(0.079)	(0.070)	(0.241)	(0.114)	(0.069)	(0.056)	(0.046)
Coca crops	0.101	−0.063	0.050	0.046	0.054	−0.038	−0.083	−0.061
	(0.111)	(0.11)	(0.090)	(0.095)	(0.094)	(0.096)	(0.099)	(0.097)
PC1	0.829***	1.169***	0.857***	1.035***	0.942***	1.154***	1.277***	1.314***
	(0.210)	(0.226)	(0.172)	(0.190)	(0.195)	(0.198)	(0.208)	(0.224)
PC5	−0.843***	−0.655**	−0.679***	−0.756***	−0.740***	−0.634**	−0.651**	−0.620**
	(0.167)	(0.232)	(0.140)	(0.177)	(0.163)	(0.203)	(0.215)	(0.210)
PC7	0.497***	0.573***	0.423***	0.468***	0.449***	0.554***	0.596***	0.584***
	(0.126)	(0.129)	(0.104)	(0.102)	(0.105)	(0.113)	(0.115)	(0.111)
PC9	−0.478**	−0.441**	−0.397**	−0.385**	−0.399**	−0.413**	−0.438**	−0.397**
	(0.161)	(0.152)	(0.132)	(0.133)	(0.134)	(0.133)	(0.138)	(0.133)
PC12	0.369*	0.371*	0.360**	0.394**	0.386**	0.374**	0.387**	0.396**
	(0.153)	(0.147)	(0.125)	(0.123)	(0.125)	(0.129)	(0.132)	(0.126)
PC13	−0.512**	−0.433**	−0.374**	−0.390**	−0.388**	−0.409**	−0.441***	−0.416***
	(0.152)	(0.147)	(0.125)	(0.125)	(0.127)	(0.128)	(0.132)	(0.126)
$\mathbf{W} \cdot y$	—	—	0.598***	—	0.426	0.361*	—	0.439*
	—	—	(0.106)	—	(0.240)	(0.157)	—	(0.182)
$\mathbf{W} \cdot u$	—	—	—	0.716***	0.406	—	−0.306	−0.532
	—	—	—	(0.093)	(0.305)	—	(0.224)	(0.325)
**W**·coca_crops	—	−0.358	—	—	—	−0.310	−0.381	−0.305
	—	(0.376)	—	—	—	(0.330)	(0.305)	(0.269)
**W**·PC1	—	−1.872**	—	—	—	−1.699**	−2.322***	−2.236***
	—	(0.676)	—	—	—	(0.592)	(0.565)	(0.584)
**W**·PC5	—	0.859	—	—	—	0.876	1.038*	1.089**
	—	(0.533)	—	—	—	(0.468)	(0.447)	(0.406)
**W**·PC7	—	−0.015	—	—	—	−0.134	−0.185	−0.390
	—	(0.379)	—	—	—	(0.346)	(0.309)	(0.303)
**W**·PC9	—	−0.294	—	—	—	−0.062	−0.275	0.013
	—	(0.427)	—	—	—	(0.389)	(0.354)	(0.307)
**W**·PC12	—	−0.632	—	—	—	−0.515	−0.785*	−0.657*
	—	(0.442)	—	—	—	(0.387)	(0.356)	(0.320)
**W**·PC13	—	−0.489	—	—	—	−0.181	−0.526	−0.152
	—	(0.489)	—	—	—	(0.446)	(0.410)	(0.377)
R^2	0.481	0.632	—	—	—	—	—	
Adj. R^2	0.429	0.551	—	—	—	—	—	
Num. obs.	78	78	78	78	78	78	78	78
Parameters	—	—	10	10	11	17	17	18
σ^2	—	—	0.376	0.362	0.372	0.343	0.356	0.319
Pseudo R^2	—	—	0.593	0.593	0.601	0.645	0.635	0.655
Wald test	—	—	31.816***	59.517***	1.776	5.312*	1.869	2.678
LogLik (Linear)	—	—	−84.579	−84.579	−84.579	−71.146	−71.146	−84.579
LogLik (Spatial)	—	—	−75.106	−75.147	−74.298	−69.779	−70.875	−68.710
AIC (Linear)	187.158	187.158	187.158	187.158	187.158	174.293	174.293	187.158
AIC (Spatial)	—	174.29	170.211	170.294	170.596	173.557	175.750	173.420
LR test	—	—	18.947***	18.865***	20.563***	2.736	0.542	31.738***

*** $p < 0.001$; ** $p < 0.01$; * $p < 0.05$

Useful links

The municipal data set used in the paper can be found at the following link:
https://www.kaggle.com/datasets/perlarivadeneyra/dataset.
All the codes to replicate the results in the paper are provided in the following dedicated repositories.

For the national level analysis:
https://www.kaggle.com/code/perlarivadeneyra/pca-all-v2
For the analysis in region A:
https://www.kaggle.com/code/perlarivadeneyra/pca-regression-region-a-v2
For the analysis in region B:
https://www.kaggle.com/code/perlarivadeneyra/pca-regression-region-b-v2
For the analysis in region C:
https://www.kaggle.com/code/perlarivadeneyra/pca-regression-region-c-v2
For the analysis in region D:
https://www.kaggle.com/code/perlarivadeneyra/pca-regression-region-d-v2
